# Seven-up acts in neuroblasts to specify adult central complex neuron identity and initiate neuroblast decommissioning

**DOI:** 10.1242/dev.202504

**Published:** 2024-02-01

**Authors:** Noah R. Dillon, Laurina Manning, Keiko Hirono, Chris Q. Doe

**Affiliations:** Institute of Neuroscience, Howard Hughes Medical Institute, University of Oregon, Eugene, OR 97403, USA

**Keywords:** Seven-up, Temporal specification, Central complex, *Drosophila*

## Abstract

An unanswered question in neurobiology is how are diverse neuron cell types generated from a small number of neural stem cells? In the *Drosophila* larval central brain, there are eight bilateral Type 2 neuroblast (T2NB) lineages that express a suite of early temporal factors followed by a different set of late temporal factors and generate the majority of the central complex (CX) neurons. The early-to-late switch is triggered by the orphan nuclear hormone receptor Seven-up (Svp), yet little is known about how this Svp-dependent switch is involved in specifying CX neuron identities. Here, we: (1) birth date the CX neurons P-EN and P-FN (early and late, respectively); (2) show that Svp is transiently expressed in all early T2NBs; and (3) show that loss of Svp expands the population of early born P-EN neurons at the expense of late born P-FN neurons. Furthermore, in the absence of Svp, T2NBs fail decommissioning and abnormally extend their lineage into week-old adults. We conclude that Svp is required to specify CX neuron identity, as well as to initiate T2NB decommissioning.

## Teaser

**Summary:** CRISPR/Cas9 knockout lines removing *svp* specifically from T2NB lineages show that Svp is required for the late born P-FN fate while restricting the early born P-EN fate.

## INTRODUCTION

Developing a complex brain requires neural stem cells to generate both a large and diverse set of neuron subtypes. *Drosophila* neuroblasts (NBs) are neural stem cells that generate neuronal diversity through the initiation of spatial patterning that establishes lineage identities (Skeath and Thor, 2003; Erclik et al., 2017) and through the subsequent temporal patterning within a lineage to produce unique neuron subtypes (Doe, 2017; El-Danaf et al., 2023). These processes have been extensively studied in embryonic ventral nerve cord NB lineages (Isshiki et al., 2001; Novotny et al., 2002; Pearson and Doe, 2003; Tran and Doe, 2008; Moris-Sanz et al., 2014; Grosskortenhaus et al., 2005, 2006), but the role of temporal patterning mechanisms in larval central brain NB lineages remains understudied.

**Fig. 1.**
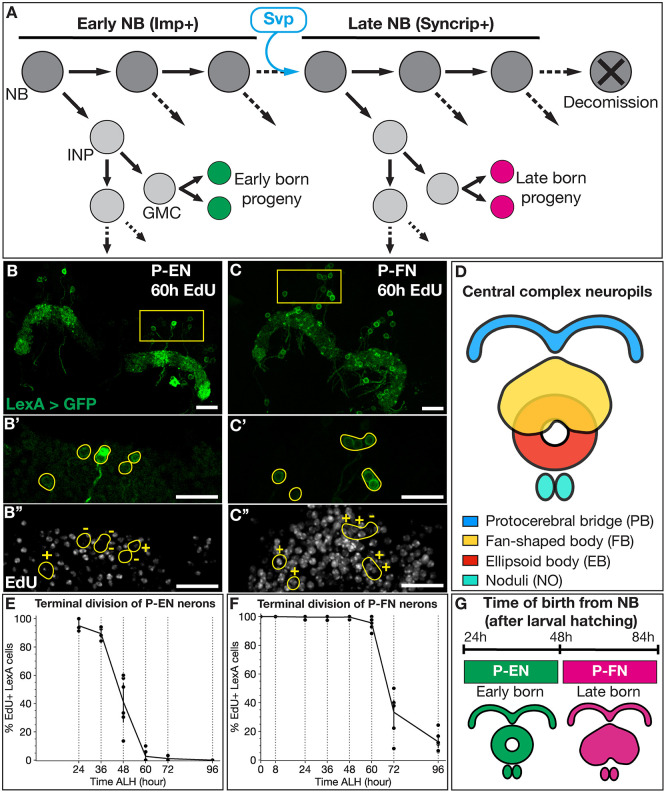
**Columnar neuron subtypes are born from larval T2NBs at distinct temporal windows.** (A) T2NBs express Imp during early larval stages and transition to Syncrip expression in late larvae due to Svp expression (Ren et al., 2017). All non-mushroom body NBs enter decommissioning in the early pupae (Ito and Hotta, 1992; Maurange et al., 2008; Siegrist et al., 2010; Homem et al., 2014; Yang et al., 2017). GMC, ganglion mother cell; INP, intermediate neural progenitor; NB, neuroblast. (B-C″) P-EN (B-B″) and P-FN (C-C″) adult neurons from EdU initiated feeding at 60 h ALH. Neurons are in green; EdU is in white. +, EdU-positive neuron; −, EdU-negative neuron. Yellow outlines in B′,B″,C′,C″ indicate cell bodies. Areas outlined in B and C are shown in more detail in images in B′,B″ and C′,C″, respectively. (D) Schematic of central complex neuropils of the adult brain. (E,F) Quantification of EdU drop out for P-EN neurons (E) and P-FN neurons (F) shown by percentage of neurons labeled by EdU. Each dot represents one adult brain. Error bars represent 95% confidence intervals. For both P-EN and P-FN neurons at each timepoint, *n*=3-7 brains. (G) Summary of P-EN and P-FN birth windows from larval T2NBs. Schematic of their neuropil targeting is shown. Scale bars: 5 µm.

The larval central brain contains ∼100 NBs per hemibrain with spatially stereotyped lineages (Pereanu and Hartenstein, 2006). Type 2 neuroblast (T2NB) lineages are generated by eight T2NBs in each hemibrain, with unique spatially defined lineage identities (Pereanu and Hartenstein, 2006; Bello et al., 2008; Boone and Doe, 2008; Bowman et al., 2008). Lineage tracing shows that each T2NB lineage (DM1-6 and DL1-2) produces neurons with lineage-specific morphology (Riebli et al., 2013; Yang et al., 2013; Andrade et al., 2019). T2NBs generate a series of intermediate neural progenitors (INPs), and each INP produces four to six ganglion mother cells, which each terminally divide to produce two post-mitotic neurons/glia (Fig. 1A). Notably, the T2NB division pattern is analogous to outer subventricular zone lineages in the primate cortex (Holguera and Desplan, 2018).

The T2NBs have been shown to express several genes in a temporal-specific manner during larval stages. The IGF-II mRNA-binding protein (Imp) is expressed in a temporal gradient in T2NBs and other lineages, with high levels of Imp in early NBs and low levels in late NBs; this is the opposite of the low-to-high temporal gradient of the RNA-binding protein Syncrip (Liu et al., 2015; Ren et al., 2017; Syed et al., 2017). Previous work has identified the orphan nuclear hormone receptor Seven-up (Svp) as a switching factor to initiate this Imp-to-Syncrip transition within T2NBs (Fig. 1A) (Ren et al., 2017; Syed et al., 2017). Similarly, Svp in ventral nerve cord NB lineages is required to switch Type 1 NBs from producing early born neuron fates to producing late born fates (Kanai et al., 2005; Mettler et al., 2006; Benito-Sipos et al., 2011; Kohwi et al., 2011). Although Svp is required to switch from early to late temporal gene expression in T2NBs, it is unknown whether this has any effect on the specification of post-mitotic neurons.

T2NB lineages generate the majority of the central complex (CX) of the *Drosophila* adult brain, with recent connectomes showing the CX containing hundreds of morphologically distinct neuron subtypes (Franconville et al., 2018; Hulse et al., 2021). One group of interest is columnar neurons, which are members of neural circuits responsible for locomotion and spatial navigation behaviors that are integrated in the CX (Giraldo et al., 2018; Green et al., 2019; Turner-Evans et al., 2020). Columnar neurons target specific neuropils of the CX (Fig. 1D) and are named according to their dendrite-axon targeting to these regions (Wolff et al., 2015; Wolff and Rubin, 2018). For example, in this study we looked at the P-EN neurons, which are named according to their dendritic targeting to the protocerebral bridge (PB) and outputs to the ellipsoid body (EB) and noduli (NO). P-FN neurons are similarly named according to their dendritic targeting to the PB and outputs to the fan-shaped body (FB) and NO. Understanding the development of T2NB lineages may shed light on how complex circuits form and drive behavior.

Here, we characterize the function of Svp in specifying post-mitotic neuron identities within T2NB lineages. We find the birth windows of P-EN and P-FN columnar neurons are from early and late T2NBs, respectively. We find that Svp is transiently and asynchronously expressed in all eight larval T2NB lineages. We used CRISPR/Cas9 knockout lines to remove *svp* specifically from T2NB lineages and show that Svp is required for the late born P-FN fate while restricting the early born P-EN fate. Additionally, we discover a previously unreported role for Svp in terminating T2NB neurogenesis. We propose that Svp is essential for early-to-late transition in T2NB lineages, and that these changes propagate down to the level of altered identity in post-mitotic neurons. Finally, we document a previously unreported function for Svp in promoting T2NB decommissioning.

## RESULTS

### Columnar neurons P-EN and P-FN are born from larval T2NBs in different temporal windows

Previous birth dating analysis of columnar neuron subtypes only assayed a subset of neurons, e.g. only 50-65% of the P-FN and P-EN neurons were birth dated (Sullivan et al., 2019). To obtain more-complete coverage, we developed a 5-ethynyl-2′-deoxyuridine (EdU) drop out approach to determine the temporal birth window for P-EN and P-FN neurons. Briefly, larvae were fed EdU, a thymidine analog that incorporates into DNA during DNA synthesis, at progressively later timepoints of larval life and maintained on EdU feeding until pupation. Thus, neurons born earlier will lose or ‘drop out’ of EdU labeling earlier than will neurons born later. We used LexA lines that specifically label the P-EN (R12D09-LexA) and P-FN (R16D01-LexA) neurons (Wolff et al., 2015; Wolff and Rubin, 2018; Sullivan et al., 2019). We found that P-EN neurons drop out from EdU labeling in early larvae by 60 h after larval hatching (ALH) (Fig. 1B-B″,E). In contrast, P-FN neurons remain EdU labeled at 60 h ALH and only drop out of EdU labeling in the late larvae by 96 h ALH (Fig. 1C-C″,F). We conclude that P-EN neurons undergo their terminal division before P-FN neurons.

We took advantage of previous cell cycle data for NBs, INPs and ganglion mother cells to provide the points at which neurons went through terminal division to when their parental INP was birthed from the NB (Homem et al., 2013). Both P-EN and P-FN neurons are derived from young INPs (Sullivan et al., 2019), and neurons from young INPs are born from their parental NB ∼12 h before terminal division (Homem et al., 2013). Therefore, we subtracted 12 h to reveal the time of origin from the T2NB. We conclude that P-EN neurons are born from T2NBs between 24 h and 48 h ALH, whereas P-FN neurons are born between 48 h and 84 h ALH (Fig. 1G). Thus, P-EN and P-FN neurons are derived from the same DM1-DM4 T2NB lineages (Yang et al., 2013) and the same young INP lineage (Sullivan et al., 2019), but differ in the timing of their birth window from the T2NB (this work). Next, we use these early and late born neuron identities to determine whether Svp, which is known for its role in regulating temporal gene expression in T2NBs (Ren et al., 2017; Syed et al., 2017), is also required for proper specification of post-mitotic neurons.

### Svp is expressed transiently and asynchronously in all larval T2NB lineages between 18 h and 24 h ALH

**Fig. 2.**
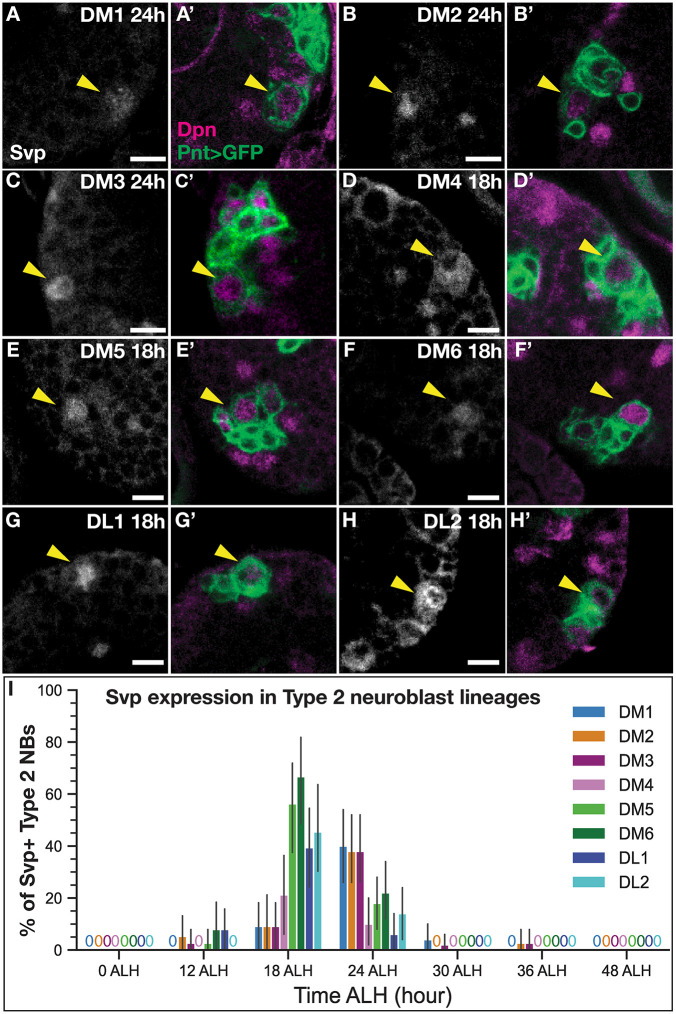
**Svp is expressed early in all larval T2NB lineages.** (A-C′) Svp is expressed 24 h after larval hatching (ALH) in T2NB lineages DM1-3. (D-H′) Svp is expressed at 18 h ALH in T2NB lineages DM4-6 and DL1-DL2. (A-H) In all images, Svp is in white and T2NBs are identified with Pnt-Gal4>GFP and Dpn. Yellow arrowheads indicate T2NB. (I) Quantification of Svp expression in T2NBs at 0 h-48 h ALH shown as a bar plot with 95% confidence interval. For each lineage, 0 ALH, *n*=34; 12 ALH, *n*=38; 18 h ALH, *n*=33; 24 h ALH, *n*=50; 30 h ALH, *n*=50; 36 h ALH, *n*=38; 48 h ALH, *n*=49 (DM lineages) or 42 (DL lineages) lobes. Scale bars: 5 µm.

Before assaying neuron identity after Svp knockout, we assayed for Svp protein expression in each of the eight T2NB lineages, primarily to confirm expression in the DM1-DM4 T2NB lineages known to generate the columnar neurons (Riebli et al., 2013; Yang et al., 2013; Andrade et al., 2019). T2NBs were identified by the co-expression of Pnt-Gal4 driving UAS membrane-bound GFP and the pan-neuroblast marker Deadpan in cells ≥5 µm in diameter. Individual T2NB lineages were identified based on the spatial position of the T2NBs within the brain lobes (Pereanu and Hartenstein, 2006; Izergina et al., 2009). We found that Svp was transiently expressed in all eight lineages with peak occurrence of expression in DM1-3 at 24 h ALH (Fig. 2A-C′,I) and for lineages DM4-6 and DL1-2 at 18 h (Fig. 2D-H′,I). Svp protein was restricted to the T2NB and absent in its progeny (Fig. 2A-H′). We saw a similar trend of Svp mRNA expression in all early T2NB lineages (Fig. S1). We conclude that Svp is transiently expressed in all T2NB lineages.

We performed Svp loss-of-function experiments with the goal of making the most complete loss- and gain-of-function alterations to the T2NB temporal factor cascade as possible, thereby increasing the chance of seeing changes in P-EN and P-FN neuron identities. We chose to knockout *svp* specifically in T2NB lineages, which has been shown to extend the expression of early NB factors (e.g. Imp and Chinmo) at the expense of late NB factors (e.g. Syncrip, EcRB1, Broad and E93) (Ren et al., 2017; Syed et al., 2017); although changes in neuronal morphology were detected, adult post-mitotic neuron molecular identity was not assayed in these experiments. We hypothesize that if early NB factors play a role in neuronal specification, then loss of Svp should result in ectopic P-EN neurons due to failure to switch to late temporal factors, and, conversely, Svp knockout should reduce or eliminate late born identities such as P-FN neurons.

We generated Svp knockouts specifically in T2NB lineages. We used two independent CRISPR/Cas9 lines (Port et al., 2020), each with two Svp-specific sgRNAs to knockout *svp* in T2NBs. To determine the efficiency of our knockouts, we tested these lines for the loss of Svp expression and recapitulation of the loss of the late T2NB expression of E93 (Syed et al., 2017). We find that our knockout lines significantly reduce the occurrence of Svp expression in T2NBs but there are a minority of escaper NBs that have expression levels indistinguishable from wild type (Fig. S2A-D); these are likely one or two T2NB lineages where CRISPR/Cas9-mediated *svp* knockout did not occur. Thus, our Svp knockouts had ‘all or none’ effects on Svp expression. T2NBs that exhibit loss of Svp also show a loss of E93 expression, as previously observed (Syed et al., 2017), further validating the Svp knockouts (Fig. S2E-H). We conclude that our CRISPR/Cas9 lines effectively knockout *svp* completely within the majority of T2NB lineages, with only a few escapers that show wild-type levels of Svp expression. We conclude that these knockout lines can be used to test the role of Svp in specifying adult columnar neuron identity.

### Cut expression distinguishes molecular identities of adult P-EN neurons from P-FN neurons

**Fig. 3.**
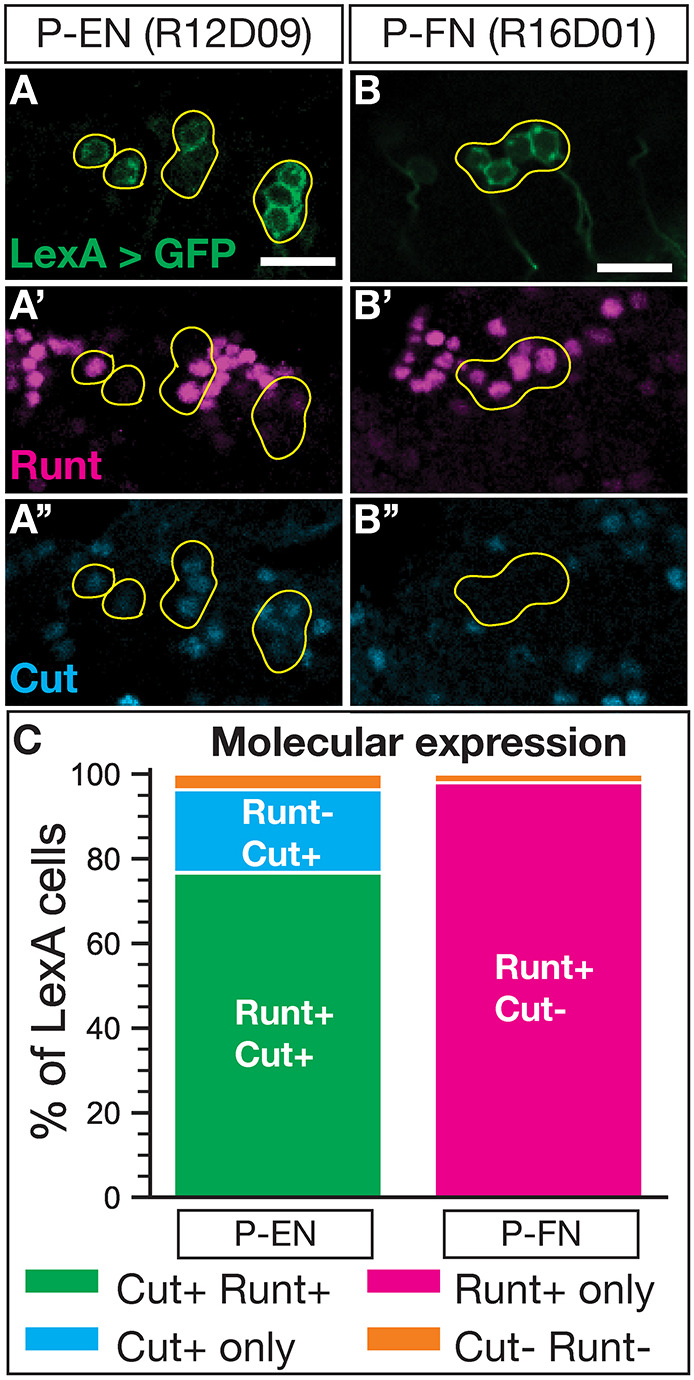
**Cut expression distinguishes P-EN and P-FN molecular identities.** (A-A″) P-EN neurons labeled using a LexA driver co-express Runt and Cut. (B-B″) P-FN neurons labeled using a LexA driver express Runt but not Cut. (C) Quantification of molecular expression in P-EN and P-FN neurons. P-EN, *n*=13 brains; P-FN, *n*=6 brains. In all panels, LexA^+^ neurons are in green, Runt is in magenta and Cut is in cyan. Neurons of interest are outlined in yellow. Scale bars: 10 µm.

P-EN and P-FN neurons were first characterized based on their distinct axon projections into CX neuropils (P-EN sends axons to the EB; P-FN sends axons to the FB) (Wolff et al., 2015). Yet to date no molecular markers have been reported to distinguish these neurons, except for the LexA lines we use here. To address this, we used a single-cell RNA-sequencing atlas of adult T2NB-derived neurons to identify previously unreported molecular markers for P-EN and P-FN neurons (Epiney et al., 2023 preprint). We identified that the homeodomain transcription factor Cut was expressed in adult P-EN neurons but not in P-FN neurons (Fig. 3A-C), whereas Svp was in neither adult neuron type (Fig. S3A-B″,C) and Runt was in both neuron types, as previously reported (Sullivan et al., 2019) (Fig. 3A′,B′; Fig. S2A-B′). We conclude that Cut distinguishes the molecular identities of adult P-EN and P-FN neurons, and we use this marker in combination with subtype-specific LexA-driven V5 and Runt expression in our analysis of the Svp knockout phenotypes.

### Loss of Svp decreases the number of late born P-FN adult neurons

**Fig. 4.**
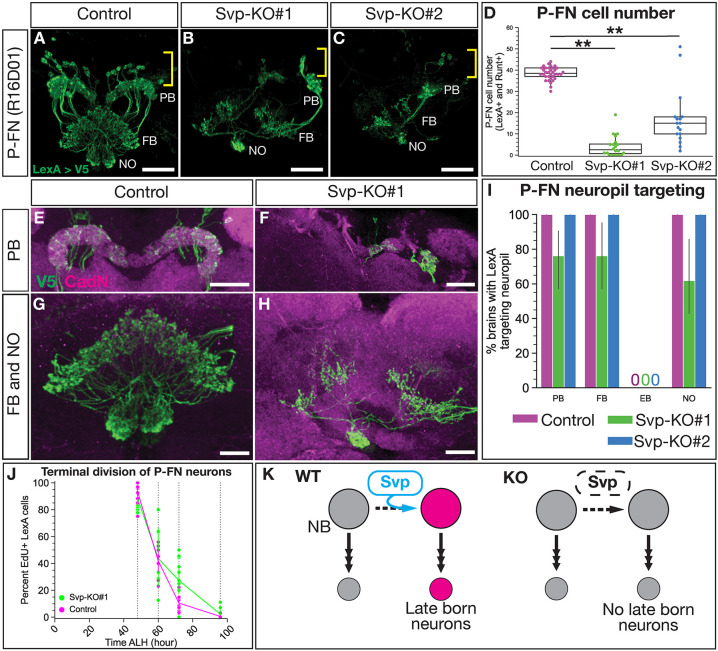
**Svp is required for the late born P-FN neuron identity.** (A-C) P-FN neurons labeled using a LexA driver expressing membrane-bound V5 show that loss of Svp leads to the loss of P-FN neurons. Yellow brackets indicate the cell body region; white text labels the neuropils: protocerebral bridge (PB), fan-shaped body (FB) and noduli (NO). (D) Quantification of P-FN cell number identified with co-expression of LexA and Runt. Each dot represents one adult brain with box and whisker plot showing distribution. Whiskers display the minimum and maximum range for the data, excluding outliers (defined as data points outside of 1.5x interquartile range). Control, *n*=34; Svp-KO#1, *n*=20; Svp-KO#2, *n*=17. *P*-values were determined using one-way ANOVA with Tukey post-hoc test. Control versus Svp-KO#1, **P<0.001; Control versus Svp-KO#2, **P<0.001. (E,F) P-FN neuron PB morphology shows lack of targeting with loss of Svp. (G,H) P-FN neuron FB and NO morphology shows lack of targeting with loss of Svp. (I) Quantification of P-FN neuropil targeting scored based on LexA targeting to neuropils identified with nc82 or CadN shown as a bar plot with 95% confidence interval. Control, *n*=35 brains; Svp-KO#1, *n*=21 brains; Svp-KO#2, *n*=13 brains; 95% confidence interval. (J) P-FN EdU dropout shows that birth of P-FN neurons in Svp-KO escaper neuroblasts occurs in a normal NB birth window, as shown by the percentage P-FN neurons labeled by EdU. Each dot represents one adult brain. Error bars represent 95% confidence intervals. For all timepoints, control, *n*=11 or 12 brains; Svp-KO#1, *n*=5-12 brains. (K) Summary of Svp required for P-FN identity. In all images, LexA^+^ neurons driving V5 are in green and CadN is in magenta. Scale bars: 20 µm in A-C; 30 µm in E; 20 µm in F; 10 µm in G,H.

Svp is required for the early-to-late switch in T2NB temporal gene expression (Ren et al., 2017; Syed et al., 2017). Here, we test whether this Svp-dependent switch in the T2NB gene expression extends to the specification of post-mitotic neurons. In this section, we ask whether Svp knockout reduces late born P-FN neurons. We used Pnt-Gal4 to drive our validated CRISPR/Cas9 lines to knock out Svp in the larval T2NB; additionally, we used a V5 membrane tag to visualize neuron specific LexA expression in the adult brain. We found Svp knockout leads to a highly penetrant loss of late born adult P-FN neurons (Fig. 4A-D). We note that there are some P-FN neurons remaining; because the cell bodies are closely clustered, they likely reflect that the Svp knockout failed to remove *svp* within individual T2NB lineages (see Discussion). Further evidence that remaining P-FN neurons derived from Svp escaper NBs include their normal morphology (Fig. 4E-I; Movies 1 and 2) and normal birth date (Fig. 4J). We conclude that Svp is required for T2NBs to produce the late born P-FN neuron identity, likely due to either an extension of early born neuron identities blocking late born identities or a failure to initiate late born identity generation (Fig. 4K).

### Loss of Svp extends the production of early born P-EN adult neurons

**Fig. 5.**
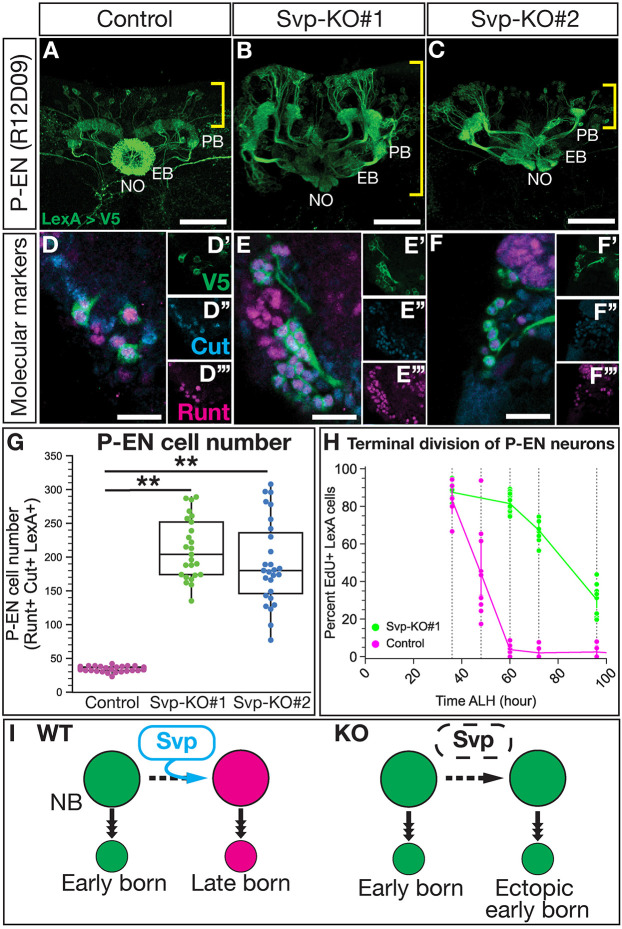
**Svp restricts the early born P-EN neuron molecular identity and birth window.** (A-C) P-FN neurons labeled using LexA drivers show loss of Svp produces ectopic P-EN neurons. Yellow brackets indicate the cell body region; white text labels neuropils: protocerebral bridge (PB), ellipsoid-body (EB) and noduli (NO). (D-F‴) Ectopic neurons maintain expression of P-EN LexA, Cut and Runt. (G) Quantification of P-EN cell numbers from A-F. Each dot represents one adult brain with box and whisker plot showing distribution. Whiskers display the minimum and maximum range for the data, excluding outliers (defined as data points outside of 1.5x interquartile range). Control, *n*=35 brains; Svp-KO#1, *n*=25 brains; Svp-KO#2, *n*=27 brains. *P*-values were determined using one-way ANOVA with Tukey's post-hoc test: control versus Svp-KO#1, **P<0.001; control versus Svp-KO#2, **P<0.001. (H) EdU dropout of P-EN neurons show an extended birth window with loss of Svp shown by percentage of P-EN neurons labeled by EdU. Each dot represents one adult brain. Error bars represent 95% confidence intervals. Control, *n*=6-10 brains; Svp-KO#1, *n*=8-13 brains. (I) Summary of Svp restricting P-EN neuron molecular identity and birth window. In all images, LexA^+^ neurons driving V5 are in green, Runt is in magenta and Cut is in cyan. Scale bars: 40 µm in A-C; 10 µm in D-F‴.

We next tested whether Svp knockout in T2NBs extends early born P-EN neuron identity, using both molecular and morphological assays. We found that loss of Svp leads to the expansion of adult P-EN neurons, with projections into the protocerebral bridge, ellipsoid body and noduli (Fig. 5A-C). Furthermore, these ectopic P-EN neurons expressed the appropriate P-EN molecular markers: P-EN LexA-driven V5, Runt and Cut (Fig. 5D-F‴,G). We conclude that Svp is required to restrict the production of P-EN neurons.

The ectopic P-EN neurons could arise from T2NBs generating more P-EN neurons at a normal early time, or they could arise from T2NBs generating P-EN neurons at abnormally late times in their lineage (replacing late born P-FN neurons). To distinguish these models, we performed EdU birth dating of P-EN neurons after Svp knockout. Whereas wild-type P-EN neurons are all born before 60 h ALH (Fig. 1B), we found that Svp knockout resulted in P-EN neurons still being born at 100 h ALH (Fig. 5H). Our data support a model in which loss of Svp extends the production of early born P-EN neurons at the expense of late born P-FN neurons (Fig. 5I).

**Fig. 6.**
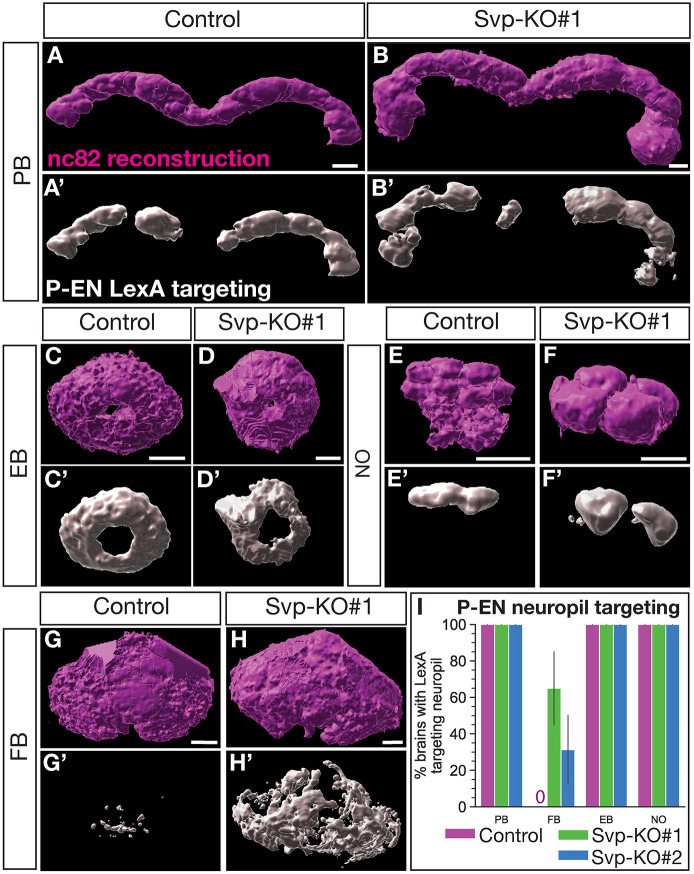
**Svp regulates the early born P-EN adult neuron morphology.** (A-H′) Reconstruction of CX neuropils and P-EN neuron targeting for the protocerebral bridge (PB; A-B′), ellipsoid body (EB; C-D′), noduli (NO; E-F′) and fan-shaped body (FB; G-H′). (I) Quantification of P-EN neuropil targeting scored based on LexA targeting to neuropils identified with nc82 or CadN shown as a bar plot with 95% confidence interval. Control, *n*=8 brains; Svp-KO#1, *n*=20 brains; Svp-KO#2, *n*=16 brains. In all panels, nc82 reconstruction is in magenta and P-EN LexA targeting is in white. Scale bars: 10 µm.

We next wanted to know whether the ectopic P-EN neurons showed normal neuropil targeting. We reconstructed CX neuropils from anti-nc82 (Bruchpilot; a presynaptic marker) (Wagh et al., 2006) stains and assayed P-EN neuropil targeting (Fig. 6; Movies 3 and 4). We first quantified all CX neuropil volumes, which includes more neuron subtypes than only the P-EN and P-FN neurons. We found that after loss of Svp in the T2NB lineages, all CX neuropils increased in size (Fig. S4A). This is due in part to ectopic P-EN neurons, which targeted all assayed CX neuropils but with increased targeting volume (Fig. 6A-H′; Fig. S4B,C). Next, we assayed neuropil targeting of ectopic P-EN neurons in our T2NB Svp knockouts. We found that ectopic P-EN neurons target all the expected CX neuropils (e.g. protocerebral bridge, ellipsoid body and noduli) but with increased volume, likely reflecting the increase in neurons (Fig. 6A-F′; Fig. S4B). We observed ectopic P-EN neurons abnormally target the fan-shaped body (Fig. 6G-H′,I, Fig. S4B). We conclude that loss of Svp leads to the generation of ectopic P-EN neurons that show targeting to normal neuropil regions with increased volume and off-targeting to the fan-shaped body.

### Loss of Svp extends T2NB lineages into the adult

**Fig. 7.**
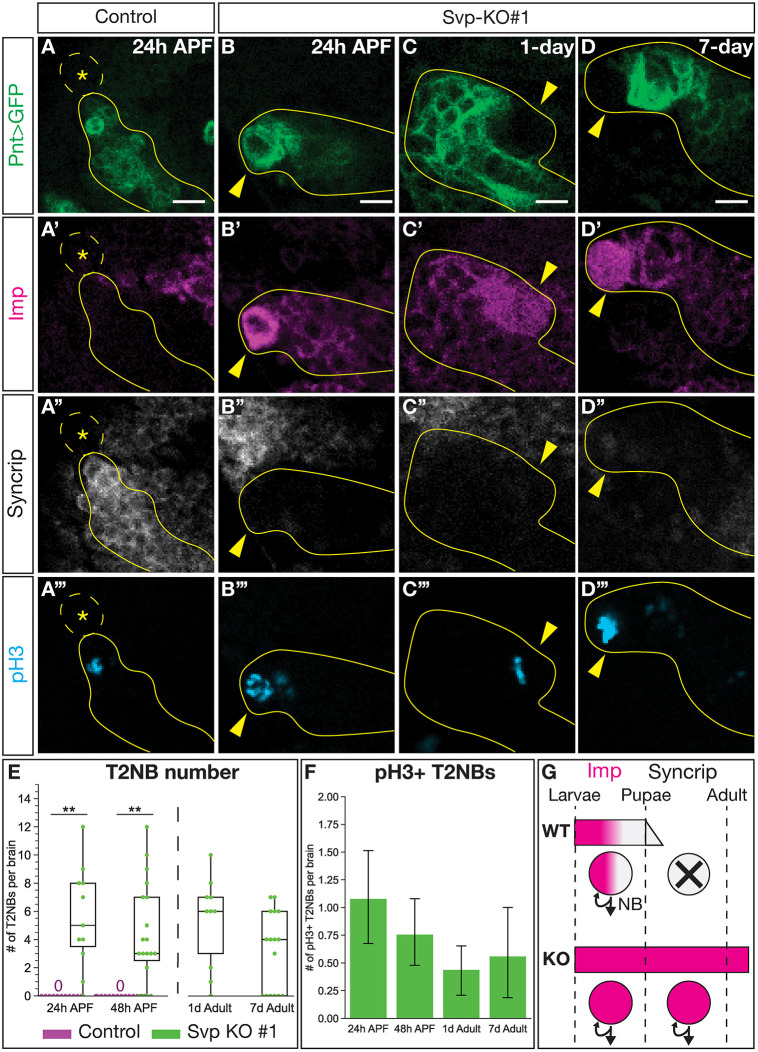
**Svp is required for timely onset of T2NB decommissioning.** (A-A‴) T2NBs have decommissioned by 24 h after pupal formation (APF). (B-D‴) Loss of Svp led to the remaining T2NBs showing expression of the early factor Imp and no expression of the late factor Syncrip, while being mitotically active with expression of pH3 at 24 h APF (B-B‴), in 1-day-old adult (C-C‴) and in 7-day-old adult (D-D‴). (A-D‴) In all images, the Type 2 lineage can be identified by Pnt-Gal4 in green, Imp in magenta, Syncrip in white and pH3 in cyan. Dashed outline and asterisk indicate lack of T2NB. Solid yellow outline indicates a type 2 lineage. Yellow arrowheads indicate T2NB. (E) Quantification of T2NB number per brain. Each dot represents one brain with box and whisker plot showing distribution. Whiskers display the minimum and maximum range for the data, excluding outliers (defined as data points outside of 1.5x interquartile range). Control 24 h APF, *n*=16 brains; Svp-KO#1 24 h APF, *n*=11 brains; control 48 h APF, *n*=18 brains; Svp-KO#1 48 h APF, *n*=19 brains; Svp-KO#1 1-day-old adult, *n*=10 brains; Svp-KO#1 7-day-old adult, *n*=16 brains. *P*-values were determined by an independent, unpaired *t*-test: control versus Svp-KO#1 at 24 h APF, **P<0.001; control versus Svp-KO#1 at 48 h APF, **P<0.001. (F) Quantification of the number of pH3^+^ ectopic T2NBs remaining in the pupae and adult stages shown as a bar plot with 95% confidence interval. Svp-KO#1 24 h APF, *n*=37 brains; Svp-KO#1 48 h APF, *n*=25 brains; Svp-KO#1 1-day-old adult, *n*=26 brains; Svp-KO#1 7-day-old adult, *n*=16 brains. (G) Summary of Svp required for transition of Imp to Syncrip expression in T2NBs for onset of neuroblast decommission. Scale bars: 5 µm.

We note above that Svp knockout extended the production of P-EN neurons beyond the later part of larval life at 100 h ALH (Fig. 5H), raising the question of whether Svp is required for terminating neurogenesis in T2NBs? Previous work has demonstrated that Svp is required for decommissioning – characterized by loss of molecular markers, cell cycle arrest, death and/or differentiation – in Type 1 NB lineages (Maurange et al., 2008; Narbonne-Reveau et al., 2016). In wild-type animals, most NBs undergo decommissioning in late larval or early pupal stages (Ito and Hotta, 1992; Maurange et al., 2008; Siegrist et al., 2010; Yang et al., 2017). The time of T2NB decommissioning has been reported to be before 24 h after pupal formation (APF) (Homem et al., 2014). We confirmed that T2NBs have completed decommissioning by 24 h (Fig. 7A-A‴,E). In contrast, Svp knockout in T2NB lineages results in delayed decommissioning with persistence of proliferative T2NBs, marked by pH3, into 7-day-old adults (Fig. 7B-D‴,E-F). Remarkably, the persistent T2NBs after Svp knockout strongly express the early marker Imp and lack the late marker Syncrip (Fig. 7B-D″), consistent with previous work showing the Imp to Syncrip gradient regulates NB decommissioning (Yang et al., 2017). Thus, we conclude that Svp is required to initiate T2NB decommissioning (Fig. 7G). How these late functions of Svp, such as T2NB decommissioning in pupal stages, are triggered by transient expression of Svp many days earlier in first instar larvae remains an interesting unanswered question.

## DISCUSSION

### Columnar neurons are born at different times in the T2NB lineage

Our EdU birth dating revealed that P-EN neurons are born from early T2NBs, and P-FN neurons are born from late T2NBs, although previous work showed P-FN neurons are born from early T2NBs (Sullivan et al., 2019). Yet there is some overlap: Sullivan et al. (2019) mapped ∼10% of P-FN neurons to a 48-60 h ALH birth window, and we birth dated most P-FN neurons to a 48-84 h ALH window (Fig. 1F,G). Importantly, our approach tracks the terminal division of cells through EdU labeling, which accounts for all neurons within the population, whereas the previous genetic approach to immortalize INPs only accounted for 50-65% of the total P-EN and P-FN neurons (Sullivan et al., 2019). We propose that EdU birth dating is more comprehensive for assigning neurons to NB birth windows, as all neurons are reliably labeled with EdU, providing reproducible tracing of terminal divisions.

It remains an unanswered question how T2NBs are patterned to generate birth order-dependent neuronal identities. One hypothesis is that the lineage uses a temporal transcription factor cascade, similar to embryonic Type 1 NBs (Isshiki et al., 2001; Novotny et al., 2002; Pearson and Doe, 2003; Tran and Doe, 2008; Moris-Sanz et al., 2014). Alternatively, or in combination, the lineage could use a temporal gradient of protein expression, such as mushroom body NBs (Liu et al., 2015). Several temporally expressed factors are known to cross-regulate in T2NBs (Ren et al., 2017; Syed et al., 2017). Our work with loss of Svp (i.e. changing expression of known temporal factors in T2NBs) shows that temporal patterning at the NB level is required to specify neuron subtypes. This is supported by recent work reporting knockdown of Imp levels in T2NB lineages results in altered CX neurons; however, it is unclear whether this is due to the role of Imp at the NB or INP level (Hamid et al., 2023 preprint). Additionally, the T2NB temporal factors EcR and E93 are required for specifying the CX neuron subtype dFB neurons (Wani et al., 2023 preprint). Our data do not distinguish between a model for a temporal transcription factor cascade and/or temporal gradients. Thus, it will be important to test individual temporal factors and expression levels in specifying birth order-dependent fates.

### Svp expression in larval T2NB lineages

We report a comprehensive characterization of Svp in all eight T2NB lineages at stages that bracket Svp expression. Previous work has only observed Svp expression at either broad temporal windows or limited to a few lineages (Bayraktar and Doe, 2013; Ren et al., 2017; Syed et al., 2017). We show that Svp has a tight expression window between 18 and 24 h ALH in all T2NB lineages, with Svp protein restricted to the NB (Fig. 2). This finding is further supported by the *svp* mRNA showing a similar expression pattern in the T2NB lineages and restricted to the NB (Fig. S1). Interestingly, *svp* mRNA was expressed before onset of protein expression and remained longer than protein expression (compare Fig. 2 to Fig. S1), which suggests post-transcriptional regulation of *svp* in the NB*.* We found evidence that indicates the posterior lineages (DM4-6, DL1-2) express Svp before the anterior lineages (DM1-3) (Fig. 2I). We speculate that the distinct onset of Svp expression between lineages may be determined by differential expression of spatial factors. Determining the upstream mechanism that initiates Svp expression remains an unanswered question. A further challenge will be resolving the orphan receptor status of Svp as it remains unknown whether Svp requires a ligand for activation.

### Svp is required for late born fates in the T2NB lineages

We found that Svp knockout resulted in a loss of the late born P-FN neuron identity. The remaining P-FN neurons resemble wild-type neurons in cell body location, numbers, morphology and birth window. We report that the Svp knockout lineages have a success rate of ∼75%, with approximately one out of four T2NBs completely escaping *svp* knockout (Fig. S2). Wild-type P-FN neurons contain ∼40 cells, but in Svp knockouts we see ∼5-15 cells remaining, which is the expected proportion based on the efficiency of our knockout lines (Fig. 4D). Moreover, the remaining P-FN neurons display lineage-appropriate morphology (Fig. 4E-H) and a wild-type birth window from the T2NBs (Fig. 4J). We conclude that the remaining P-FN neurons are derived from Svp knockout escaping NBs that maintain normal Svp expression and hence develop normally. Thus, our Svp knockout can be described as an ‘all or none’ knockout of Svp in ∼75% of the T2NBs.

### Svp specification of other CX neuron subtypes

Svp has previously been reported as either a switching factor in embryonic and larval NBs (Kanai et al., 2005; Mettler et al., 2006; Maurange et al., 2008; Benito-Sipos et al., 2011; Kohwi et al., 2011) or as a post-mitotic fate determinant in photoreceptor neuron subtypes (Mlodzik et al., 1990; Hiromi et al., 1993). Our work shows that Svp acts as a switching factor in T2NBs for specifying neuron identities, as Svp was not expressed in post-mitotic P-EN or P-FN neurons (Fig. S3). However, we cannot rule out the possibility that Svp is required as a post-mitotic fate determinant for other CX subtypes.

We show that Svp is required to restrict the early born P-EN identity, as loss of Svp resulted in T2NBs extending production of P-EN neurons into an abnormally late temporal window (Fig. 5H). These ectopic P-EN neurons maintain wild-type molecular markers (e.g. LexA, Runt and Cut) (Fig. 5G). We note that these ectopic P-EN neurons have ectopic targeting to the fan-shaped body, a neuropil not extensively targeted by wild-type P-EN neurons (Hulse et al., 2021) (Fig. 6G-H′,I; Fig. S4B,C). We speculate that this abnormal targeting may be compensation for the loss of P-FN neurons that normally target the region, suggesting that columnar neuron targeting may be promiscuous when subtypes are absent. Alternatively, the ectopic targeting to the fan-shaped body may be an expanded volume of neurites from the increased number of P-EN neurons as they pass through to target the ellipsoid body. We note that P-EN neurons are only one fate born from the early temporal window; other neurons born at a similar time are not visible with the P-EN/P-FN LexA markers used here. We hypothesize that these early born populations will also be expanded with the loss of Svp. It will be vital for future work to identify additional CX neuron markers and their birth date from the T2NBs to test for functional temporal patterning factors.

We find that the loss of Svp resulted in significant changes to CX neuropil volumes. Although targeting from ectopic P-EN neurons accounts for some of this increase, it does not fully account for the global CX neuropil enlargement we report (Fig. S4B,C). We speculate that either expanded populations of other T2NB-derived neuron subtypes and/or synaptic partners account for the ectopic targeting to these neuropils. For example, P-EN neurons form synapses with the T2NB-derived E-PG neurons (Green et al., 2017, 2019); thus, loss of Svp could lead to either E-PG compensation with increased targeting to ectopic P-EN neurons and/or an expanded E-PG neuron population. We are unable to account for the global CX changes that occurred with the loss of Svp due to the limitation in our assays of only the P-EN and P-FN neurons.

### Svp-mediated regulation of Type 2 neuroblast temporal progression

Svp is expressed in early larval T2NBs prior to the Imp to Syncrip transition (Ren et al., 2017); thus, we were surprised by our findings that Svp is required for terminating neurogenesis in pupae. Previous work has shown the transition in central brain NBs from Imp to Syncrip expression initiates NB decommissioning (Yang et al., 2017). Interestingly, mushroom body NBs continue proliferating into pupal stages, when other NBs have decommissioned, owing to sustained Imp expression (Yang et al., 2017). Consistent with our work on T2NBs, previous work has also demonstrated that loss of Svp in the ventral nerve cord and central brain NB lineages leads to continued NB proliferation into the adult (Maurange et al., 2008; Narbonne-Reveau et al., 2016). We report that loss of Svp in early larval T2NBs had profound impacts on the lifespan of the NBs, where they survive into 7-day adults manifesting: (1) maintained Imp expression; (2) mitotic activation; and (3) maintenance of low levels of Syncrip (Fig. 7). Thus, we propose that Svp initiating the switch of Imp-to-Syncrip in T2NBs is a NB-autonomous mechanism required for terminating neurogenesis within T2NB lineages. This is consistent with previous work showing that Svp is required for the progression of Imp to Syncrip expression in T2NBs (Ren et al., 2017). Two hypotheses are that Svp initiates a transcriptional cascade that executes a function ∼24 h later, or that Svp alters the NB chromatin landscape to make it responsive to a new input ∼24 h later. It will be important future work to find the targets of Svp within the T2NBs.

Recently, Notch signaling has been shown to be required for central brain Type 1 NB decommissioning by disrupting temporal patterning progression; loss of Notch signaling produced prolonged expression of the early factor Imp and reduced expression of the late factor E93 (Sood et al., 2023). Additionally, Notch signaling appeared to be required in terminating expression of the early factors Castor and Svp (Sood et al., 2023). This indicates that Notch signaling acts upstream of NB temporal factors, and thus is also likely to act upstream of Svp. This is difficult to test because loss of Notch signaling in T2NBs results in loss of NB identity (San-Juán and Baonza, 2011; Zhu et al., 2012; Li et al., 2016, 2017).

### Conserved role of Svp in vertebrate temporal patterning

Our work shows that Svp acts as a switching factor in T2NBs to switch from producing early born to late born neuronal identities. The mammalian orthologs of *svp*, *COUP-TFI* and *COUP-TFII* (*Nr2f1* and *Nr2f2*) have been characterized for a similar role as a switching factor in murine neural stem cells (Naka et al., 2008; Lodato et al., 2011). *COUP-TFI* and *COUP-TFII* are required for cultured neural stem cells to switch from producing early born neuron cell types to producing late born glia, because a loss of this molecular switch resulted in sustained neurogenesis (Naka et al., 2008). Additionally, *COUP-TFI* and *COUP-TFII* are required for switching cortical neural stem cells from producing early born interneuron fates to late born interneuron fates (Lodato et al., 2011). These findings, along with ours and others in *Drosophila*, suggest that Svp has a conserved role as a neural stem cell switching factor from fly to mammals (Mettler et al., 2006; Benito-Sipos et al., 2011; Ren et al., 2017).

## MATERIALS AND METHODS

### Animal preparation

*Drosophila melanogaster* was used in all experiments. All flies were kept and maintained at 25°C unless stated otherwise. Stocks used can be found in Table S1 and experimental genetic crosses in Table S2.

### EdU experiments

EDU (5-ethynyl-2′-deoxyuridine; Millipore-Sigma, 900584-50MG), a thymidine analog, was used to label proliferating cells starting at various sequential larvae ages. Larvae were fed food containing 20 μg/ml EdU nonstop from the initial age feeding started until the larvae pupated. Larvae fed on EdU were raised at temperatures between 18°C and 21°C until adults hatched and were dissected.

### Larval experiments

Embryos were collected on 3% agar apple juice caps with yeast paste for 4 h and aged for 21 h. After aging, embryos were transferred to a fresh cap and aged 4 h for hatching. Hatched larvae were collected and dissected at the corresponding time after larval hatching (ALH).

### Adult experiments

Males and virgin females were introduced in standard yeast medium vials and flipped every 2 days. Adult flies (2-5 days old) were dissected for all experiments unless stated otherwise. All animals dissected were a mixture of male and female, unless otherwise specified.

### Hybridization chain reaction (HCR) RNA fluorescent *in situ* hybridization

Larval brains were dissected in Schneider's insect medium, fixed in 4% PFA (paraformaldehyde; Electron Microscopy Sciences, 15710) in PBS (phosphate-buffered saline; Sigma-Aldrich, P4417) for 7-15 min at room temperature, and washed in PBST (PBS with 0.3% Triton; Sigma-Aldrich, T8787). The fixed brains were stored in 70% ethanol in deionized water at 4°C until used. We followed the protocol from Duckhorn et al. (2022). A 20-probe set targeting all *svp* transcript isoforms was synthesized by Molecular Instruments and probes were added to a final concentration of 4 nM for hybridization. Amplifier B3-546 was also synthesized by Molecular Instruments and 6 pmol of each hairpin (h1 and h2) was added for amplification.

### Larval brain sample preparation

Larval brains were dissected in PBS and mounted on poly-D-lysine coated coverslips (Neuvitro Corporation GG-12-PDL; primed in 100% ethanol). Samples fixed for 23 min in 4% PFA in PBST. Samples were washed in PBST and blocked with 2% normal donkey serum (Jackson ImmunoResearch Laboratories) in PBST. Samples were incubated in a primary antibody mix diluted in PBST for overnight or for 1-2 days at 4°C. Primary antibodies were removed and samples thoroughly washed with PBST. Samples were incubated in secondary antibodies overnight at 4°C. Secondary antibodies were removed and samples washed in PBST. Samples were dehydrated with an ethanol series of 30%, 50%, 75% and 100% ethanol then incubated in xylene (Fisher Chemical, X5-1) for 2×10 min. Samples were mounted onto slides with DPX (Sigma-Aldrich 06552) and cured for 3-4 days then stored at 4°C until imaged.

### Adult brain sample preparation

Adult brains were prepared in a similar way to larval brains, with the exception of 41 min for fixation in 4% PFA and 2×12 min xylene incubations.

### EdU adult brain sample preparation

Adult brains from EdU-fed larvae were dissected in HL3.1 then fixed in 4% PFA for 30 min and incubated in block at 4°C overnight. Samples were incubated in primary and secondary mixes before Click-it-Reaction to label EdU. The Click-it-Reaction mix comprised PBS, Copper II sulfate (ThermoFisher, 033308.22), 555-Azide (ThermoFisher, A20012) in DSMO and ascorbic acid (Sigma-Aldrich, A4544-25G) for a 2 h incubation. Samples were dehydrated and washed in xylene before DPX mounting as described above.

### Antibodies

Antibodies used can found in Table S3.

### Confocal microscopy

Fixed preparations were imaged with a Zeiss LSM 900 or 800 laser scanning confocal equipped with an Axio Imager.Z2 microscope. A 10×/0.3 EC Plan-Neofluar M27 or 40×/1.40 NA Oil Plan-Apochromat DIC M27 objective lens was used. The software program used was Zen 3.6 (blue edition) (Zeiss AG).

### Image processing and analysis

#### Cell counting and neuropil target scoring

Confocal image stacks were loaded into Fiji (ImageJ 1.50d, https://imagej.net/Fiji). Cells were counted using the Cell Counter plug-in. Neuropil targeting was determined by colocalization of LexA expression with a neuropil marker that was not a filament bundle passing through the neuropil.

#### Imaris neuropil reconstructions

Confocal image stacks were loaded into Imaris 10.0.0 (Bitplane). Imaris Surface objects were created for each neuropil using nc82 staining and LexA expression followed by new objects designating overlap between neuropil and neurites. Briefly, the Surface tool was selected and a region of interest (ROI) was drawn to encompass a whole CX neuropil or LexA expression. The source channel was selected (nc82 in RRX for neuropils or LexA in 647 for neurites) and absolute threshold intensity was manually set slice by slice to outline fluorescent signal and morphologically split to separate regions. All Starting Points and Seed Points were kept, ensuring full coverage of signal. Surfaces were rendered and surfaces outside neuropil structures removed. To find the LexA targeting for each neuropil, Surface-Surface Overlap File XTension (Matthew Gastinger; https://imaris.oxinst.com/open/view/surface-surface-overlap) was used to find the volume (µm^3^) of overlap. A Smoothing Factor of 0.2 µm was kept for all surfaces.

#### Figure preparation

Images in figures were prepared either in Imaris 10.0.0 or Fiji. Scale bars are given for a single slice in all single slice images and from all stacks within maximum intensity projections images. Pixel brightness was adjusted in images for clearer visualization; all adjustments were made uniformly over the entire image, and uniformly across wild-type samples and corresponding control and experimental samples. Adobe Illustrator 2023 was used for formatting.

### Statistical analyses

Statistics were computed using Python tests (see https://github.com/nrdDrosophila/Seven-up-specifies-neuron-identity). All statistical tests used are listed in the figure legends. *P*-values are reported in the figure legends. Plots display n.s.=not significant, *P<0.05 and **P<0.01. Plots were generated using Seaborn and Matplotlib packages in Python. 95% confidence intervals and boxplot distributions were calculated when plotting the data.

## Supplementary Material

10.1242/develop.202504_sup1 Supplementary information

## References

[DEV202504C1] Andrade, I. V., Riebli, N., Nguyen, B.-C. M., Omoto, J. J., Cardona, A. and Hartenstein, V. (2019). Developmentally arrested precursors of pontine neurons establish an embryonic blueprint of the Drosophila central complex. *Curr. Biol.* 29, 412-425.e3. 10.1016/j.cub.2018.12.012 30661802 PMC6524766

[DEV202504C2] Bayraktar, O. A. and Doe, C. Q. (2013). Combinatorial temporal patterning in progenitors expands neural diversity. *Nature* 498, 449-455. 10.1038/nature12266 23783519 PMC3941985

[DEV202504C3] Bello, B. C., Izergina, N., Caussinus, E. and Reichert, H. (2008). Amplification of neural stem cell proliferation by intermediate progenitor cells in Drosophila brain development. *Neural Dev.* 3, 5. 10.1186/1749-8104-3-5 18284664 PMC2265709

[DEV202504C4] Benito-Sipos, J., Ulvklo, C., Gabilondo, H., Baumgardt, M., Angel, A., Torroja, L. and Thor, S. (2011). Seven up acts as a temporal factor during two different stages of neuroblast 5-6 development. *Development* 138, 5311-5320. 10.1242/dev.070946 22071101

[DEV202504C5] Boone, J. Q. and Doe, C. Q. (2008). Identification of Drosophila type II neuroblast lineages containing transit amplifying ganglion mother cells. *Dev. Neurobiol.* 68, 1185-1195. 10.1002/dneu.20648 18548484 PMC2804867

[DEV202504C6] Bowman, S. K., Rolland, V., Betschinger, J., Kinsey, K. A., Emery, G. and Knoblich, J. A. (2008). The tumor suppressors brat and numb regulate transit-amplifying neuroblast lineages in Drosophila. *Dev. Cell* 14, 535-546. 10.1016/j.devcel.2008.03.004 18342578 PMC2988195

[DEV202504C7] Doe, C. Q. (2017). Temporal patterning in the Drosophila CNS. *Annu. Rev. Cell Dev. Biol.* 33, 219-240. 10.1146/annurev-cellbio-111315-125210 28992439

[DEV202504C8] Duckhorn, J. C., Junker, I. P., Ding, Y. and Shirangi, T. R. (2022). Combined in situ hybridization chain reaction and immunostaining to visualize gene expression in whole-mount Drosophila central nervous systems. In *Behavioral Neurogenetics* (ed. D. Yamamoto), pp. 1-14. New York, NY: Springer US.

[DEV202504C9] El-Danaf, R. N., Rajesh, R. and Desplan, C. (2023). Temporal regulation of neural diversity in Drosophila and vertebrates. *Semin. Cell Dev. Biol.* 142, 13-22. 10.1016/j.semcdb.2022.05.011 35623984 PMC11585012

[DEV202504C10] Epiney, D., Chaya, G. M., Dillon, N., Lai, S.-L. and Doe, C. (2023). Transcriptional complexity in the insect central complex: single nuclei RNA sequencing of adult brain neurons derived from type 2 neuroblasts. *bioRxiv* 2023.12.10.571022. 10.1101/2023.12.10.571022

[DEV202504C11] Erclik, T., Li, X., Courgeon, M., Bertet, C., Chen, Z., Baumert, R., Ng, J., Koo, C., Arain, U., Behnia, R. et al. (2017). Integration of temporal and spatial patterning generates neural diversity. *Nature* 541, 365-370. 10.1038/nature20794 28077877 PMC5489111

[DEV202504C12] Franconville, R., Beron, C. and Jayaraman, V. (2018). Building a functional connectome of the Drosophila central complex. *eLife* 7, e37017. 10.7554/eLife.37017 30124430 PMC6150698

[DEV202504C13] Giraldo, Y. M., Leitch, K. J., Ros, I. G., Warren, T. L., Weir, P. T. and Dickinson, M. H. (2018). Sun navigation requires compass neurons in Drosophila. *Curr. Biol.* 28, 2845-2852.e4. 10.1016/j.cub.2018.07.002 30174187 PMC7301569

[DEV202504C14] Green, J., Adachi, A., Shah, K. K., Hirokawa, J. D., Magani, P. S. and Maimon, G. (2017). A neural circuit architecture for angular integration in Drosophila. *Nature* 546, 101-106. 10.1038/nature22343 28538731 PMC6320684

[DEV202504C15] Green, J., Vijayan, V., Mussells Pires, P., Adachi, A. and Maimon, G. (2019). A neural heading estimate is compared with an internal goal to guide oriented navigation. *Nat. Neurosci.* 22, 1460-1468. 10.1038/s41593-019-0444-x 31332373 PMC7688015

[DEV202504C16] Grosskortenhaus, R., Pearson, B. J., Marusich, A. and Doe, C. Q. (2005). Regulation of temporal identity transitions in Drosophila neuroblasts. *Dev. Cell* 8, 193-202. 10.1016/j.devcel.2004.11.019 15691761

[DEV202504C17] Grosskortenhaus, R., Robinson, K. J. and Doe, C. Q. (2006). Pdm and Castor specify late-born motor neuron identity in the NB7-1 lineage. *Genes Dev.* 20, 2618-2627. 10.1101/gad.1445306 16980589 PMC1578683

[DEV202504C18] Hamid, A., Gattuso, H., Caglar, A. N., Pillai, M., Steele, T., Gonzalez, A., Nagel, K. and Syed, M. H. (2023). The RNA-binding protein, Imp specifies olfactory navigation circuitry and behavior in Drosophila. *bioRxiv* 2023.05.26.542522. 10.1101/2023.05.26.542522

[DEV202504C19] Hiromi, Y., Mlodzik, M., West, S. R., Rubin, G. M. and Goodman, C. S. (1993). Ectopic expression of seven-up causes cell fate changes during ommatidial assembly. *Development* 118, 1123-1135. 10.1242/dev.118.4.1123 8269843

[DEV202504C20] Holguera, I. and Desplan, C. (2018). Neuronal specification in space and time. *Science* 362, 176-180. 10.1126/science.aas9435 30309944 PMC6368964

[DEV202504C21] Homem, C. C. F., Reichardt, I., Berger, C., Lendl, T. and Knoblich, J. A. (2013). Long-term live cell imaging and automated 4D analysis of Drosophila neuroblast lineages. *PLoS ONE* 8, e79588. 10.1371/journal.pone.0079588 24260257 PMC3832664

[DEV202504C22] Homem, C. C. F., Steinmann, V., Burkard, T. R., Jais, A., Esterbauer, H. and Knoblich, J. A. (2014). Ecdysone and mediator change energy metabolism to terminate proliferation in Drosophila neural stem cells. *Cell* 158, 874-888. 10.1016/j.cell.2014.06.024 25126791

[DEV202504C23] Hulse, B. K., Haberkern, H., Franconville, R., Turner-Evans, D. B., Takemura, S., Wolff, T., Noorman, M., Dreher, M., Dan, C., Parekh, R. et al. (2021). A connectome of the Drosophila central complex reveals network motifs suitable for flexible navigation and context-dependent action selection. *eLife* 10, e66039. 10.7554/eLife.66039 34696823 PMC9477501

[DEV202504C24] Isshiki, T., Pearson, B., Holbrook, S. and Doe, C. Q. (2001). Drosophila neuroblasts sequentially express transcription factors which specify the temporal identity of their neuronal progeny. *Cell* 106, 511-521. 10.1016/S0092-8674(01)00465-2 11525736

[DEV202504C25] Ito, K. and Hotta, Y. (1992). Proliferation pattern of postembryonic neuroblasts in the brain of Drosophila melanogaster. *Dev. Biol.* 149, 134-148. 10.1016/0012-1606(92)90270-Q 1728583

[DEV202504C26] Izergina, N., Balmer, J., Bello, B. and Reichert, H. (2009). Postembryonic development of transit amplifying neuroblast lineages in the Drosophila brain. *Neural Dev.* 4, 44. 10.1186/1749-8104-4-44 20003348 PMC2801669

[DEV202504C27] Kanai, M. I., Okabe, M. and Hiromi, Y. (2005). seven-up controls switching of transcription factors that specify temporal identities of Drosophila neuroblasts. *Dev. Cell* 8, 203-213. 10.1016/j.devcel.2004.12.014 15691762

[DEV202504C28] Kohwi, M., Hiebert, L. S. and Doe, C. Q. (2011). The pipsqueak-domain proteins Distal antenna and Distal antenna-related restrict Hunchback neuroblast expression and early-born neuronal identity. *Development* 138, 1727-1735. 10.1242/dev.061499 21429984 PMC3074449

[DEV202504C29] Li, X., Xie, Y. and Zhu, S. (2016). Notch maintains *Drosophila* type II neuroblasts by suppressing the expression of the Fez transcription factor Earmuff. *Development* 143, 2511-2521. 10.1242/dev.136184 27151950 PMC4958340

[DEV202504C30] Li, X., Chen, R. and Zhu, S. (2017). bHLH-O proteins balance the self-renewal and differentiation of Drosophila neural stem cells by regulating Earmuff expression. *Dev. Biol.* 431, 239-251. 10.1016/j.ydbio.2017.09.011 28899667 PMC5658246

[DEV202504C31] Liu, Z., Yang, C.-P., Sugino, K., Fu, C.-C., Liu, L.-Y., Yao, X., Lee, L. P. and Lee, T. (2015). Opposing intrinsic temporal gradients guide neural stem cell production of varied neuronal fates. *Science* 350, 317-320. 10.1126/science.aad1886 26472907

[DEV202504C32] Lodato, S., Tomassy, G. S., De Leonibus, E., Uzcategui, Y. G., Andolfi, G., Armentano, M., Touzot, A., Gaztelu, J. M., Arlotta, P., Menendez de la Prida, L. et al. (2011). Loss of COUP-TFI alters the balance between caudal ganglionic eminence- and medial ganglionic eminence-derived cortical interneurons and results in resistance to epilepsy. *J. Neurosci.* 31, 4650-4662. 10.1523/JNEUROSCI.6580-10.2011 21430164 PMC6622915

[DEV202504C33] Maurange, C., Cheng, L. and Gould, A. P. (2008). Temporal transcription factors and their targets schedule the end of neural proliferation in Drosophila. *Cell* 133, 891-902. 10.1016/j.cell.2008.03.034 18510932

[DEV202504C34] Mettler, U., Vogler, G. and Urban, J. (2006). Timing of identity: spatiotemporal regulation of hunchback in neuroblast lineages of Drosophila by Seven-up and Prospero. *Development* 133, 429-437. 10.1242/dev.02229 16396905

[DEV202504C35] Mlodzik, M., Hiromi, Y., Weber, U., Goodman, C. S. and Rubin, G. M. (1990). The Drosophila seven-up gene, a member of the steroid receptor gene superfamily, controls photoreceptor cell fates. *Cell* 60, 211-224. 10.1016/0092-8674(90)90737-Y 2105166

[DEV202504C36] Moris-Sanz, M., Estacio-Gómez, A., Álvarez-Rivero, J. and Díaz-Benjumea, F. J. (2014). Specification of neuronal subtypes by different levels of Hunchback. *Development* 141, 4366-4374. 10.1242/dev.113381 25344076

[DEV202504C37] Naka, H., Nakamura, S., Shimazaki, T. and Okano, H. (2008). Requirement for COUP-TFI and II in the temporal specification of neural stem cells in CNS development. *Nat. Neurosci.* 11, 1014-1023. 10.1038/nn.2168 19160499

[DEV202504C38] Narbonne-Reveau, K., Lanet, E., Dillard, C., Foppolo, S., Chen, C.-H., Parrinello, H., Rialle, S., Sokol, N. S. and Maurange, C. (2016). Neural stem cell-encoded temporal patterning delineates an early window of malignant susceptibility in Drosophila. *eLife* 5, e13463. 10.7554/eLife.13463 27296804 PMC4907696

[DEV202504C39] Novotny, T., Eiselt, R. and Urban, J. (2002). Hunchback is required for the specification of the early sublineage of neuroblast 7-3 in the Drosophila central nervous system. *Development* 129, 1027-1036. 10.1242/dev.129.4.1027 11861485

[DEV202504C40] Pearson, B. J. and Doe, C. Q. (2003). Regulation of neuroblast competence in Drosophila. *Nature* 425, 624-628. 10.1038/nature01910 14534589

[DEV202504C41] Pereanu, W. and Hartenstein, V. (2006). Neural lineages of the Drosophila brain: a three-dimensional digital atlas of the pattern of lineage location and projection at the late larval stage. *J. Neurosci.* 26, 5534-5553. 10.1523/JNEUROSCI.4708-05.2006 16707805 PMC6675312

[DEV202504C42] Port, F., Strein, C., Stricker, M., Rauscher, B., Heigwer, F., Zhou, J., Beyersdörffer, C., Frei, J., Hess, A., Kern, K. et al. (2020). A large-scale resource for tissue-specific CRISPR mutagenesis in Drosophila. *eLife* 9, e53865. 10.7554/eLife.53865 32053108 PMC7062466

[DEV202504C43] Ren, Q., Yang, C.-P., Liu, Z., Sugino, K., Mok, K., He, Y., Ito, M., Nern, A., Otsuna, H. and Lee, T. (2017). Stem cell-intrinsic, seven-up-triggered temporal factor gradients diversify intermediate neural progenitors. *Curr. Biol.* 27, 1303-1313. 10.1016/j.cub.2017.03.047 28434858

[DEV202504C44] Riebli, N., Viktorin, G. and Reichert, H. (2013). Early-born neurons in type II neuroblast lineages establish a larval primordium and integrate into adult circuitry during central complex development in Drosophila. *Neural Dev.* 8, 6. 10.1186/1749-8104-8-6 23618231 PMC3685605

[DEV202504C45] San-Juán, B. P. and Baonza, A. (2011). The bHLH factor deadpan is a direct target of Notch signaling and regulates neuroblast self-renewal in Drosophila. *Dev. Biol.* 352, 70-82. 10.1016/j.ydbio.2011.01.019 21262215

[DEV202504C46] Siegrist, S. E., Haque, N. S., Chen, C.-H., Hay, B. A. and Hariharan, I. K. (2010). Inactivation of both foxo and reaper promotes long-term adult neurogenesis in Drosophila. *Curr. Biol.* 20, 643-648. 10.1016/j.cub.2010.01.060 20346676 PMC2862284

[DEV202504C47] Skeath, J. B. and Thor, S. (2003). Genetic control of Drosophila nerve cord development. *Curr. Opin. Neurobiol.* 13, 8-15. 10.1016/S0959-4388(03)00007-2 12593977

[DEV202504C48] Sood, C., Nahid, M. A., Branham, K. R., Pahl, M. C., Doyle, S. E. and Siegrist, S. E. (2023). Delta-dependent Notch activation closes the early neuroblast temporal program to promote lineage progression and neurogenesis termination in Drosophila. *eLife* 12. 10.7554/eLife.88565.1 PMC10942576 38391176

[DEV202504C49] Sullivan, L. F., Warren, T. L. and Doe, C. Q. (2019). Temporal identity establishes columnar neuron morphology, connectivity, and function in a Drosophila navigation circuit. *eLife* 8, e43482. 10.7554/eLife.43482 30706848 PMC6386519

[DEV202504C50] Syed, M. H., Mark, B. and Doe, C. Q. (2017). Steroid hormone induction of temporal gene expression in Drosophila brain neuroblasts generates neuronal and glial diversity. *eLife* 6, e26287. 10.7554/eLife.26287 28394252 PMC5403213

[DEV202504C51] Tran, K. D. and Doe, C. Q. (2008). Pdm and Castor close successive temporal identity windows in the NB3-1 lineage. *Development* 135, 3491-3499. 10.1242/dev.024349 18832394 PMC3989073

[DEV202504C52] Turner-Evans, D. B., Jensen, K. T., Ali, S., Paterson, T., Sheridan, A., Ray, R. P., Wolff, T., Lauritzen, J. S., Rubin, G. M., Bock, D. D. et al. (2020). The neuroanatomical ultrastructure and function of a biological ring attractor. *Neuron* 108, 145-163.e10. 10.1016/j.neuron.2020.08.006 32916090 PMC8356802

[DEV202504C53] Wagh, D. A., Rasse, T. M., Asan, E., Hofbauer, A., Schwenkert, I., Dürrbeck, H., Buchner, S., Dabauvalle, M.-C., Schmidt, M., Qin, G. et al. (2006). Bruchpilot, a protein with homology to ELKS/CAST, is required for structural integrity and function of synaptic active zones in Drosophila. *Neuron* 49, 833-844. 10.1016/j.neuron.2006.02.008 16543132

[DEV202504C54] Wani, A. R., Chowdhury, B., Luong, J., Chaya, G. M., Patel, K., Isaacman-Beck, J., Shafer, O., Kayser, M. S. and Syed, M. H. (2023). Stem cell-specific ecdysone signaling regulates the development and function of a Drosophila sleep homeostat. *bioRxiv* 2023.09.29.560022. 10.1101/2023.09.29.560022

[DEV202504C55] Wolff, T. and Rubin, G. M. (2018). Neuroarchitecture of the Drosophila central complex: a catalog of nodulus and asymmetrical body neurons and a revision of the protocerebral bridge catalog. *J. Comp. Neurol.* 526, 2585-2611. 10.1002/cne.24512 30084503 PMC6283239

[DEV202504C56] Wolff, T., Iyer, N. A. and Rubin, G. M. (2015). Neuroarchitecture and neuroanatomy of the Drosophila central complex: A GAL4-based dissection of protocerebral bridge neurons and circuits. *J. Comp. Neurol.* 523, 997-1037. 10.1002/cne.23705 25380328 PMC4407839

[DEV202504C57] Yang, J. S., Awasaki, T., Yu, H.-H., He, Y., Ding, P., Kao, J.-C. and Lee, T. (2013). Diverse neuronal lineages make stereotyped contributions to the Drosophila locomotor control center, the central complex. *J. Comp. Neurol.* 521, 2645-2662. 10.1002/cne.23339 23696496 PMC3902843

[DEV202504C58] Yang, C.-P., Samuels, T. J., Huang, Y., Yang, L., Ish-Horowicz, D., Davis, I. and Lee, T. (2017). Imp and Syp RNA-binding proteins govern decommissioning of Drosophila neural stem cells. *Development* 144, 3454-3464. 10.1242/dev.149500 28851709 PMC5665480

[DEV202504C59] Zhu, S., Wildonger, J., Barshow, S., Younger, S., Huang, Y. and Lee, T. (2012). The bHLH repressor deadpan regulates the self-renewal and specification of Drosophila larval neural stem cells independently of Notch. *PLoS ONE* 7, e46724. 10.1371/journal.pone.0046724 23056424 PMC3466283

