# Supplementary material for: Seven-up acts in neuroblasts to specify adult central complex neuron identity and initiate neuroblast decommissioning
Source: Development. 2024 Feb 1;151(3):dev202504. doi: 10.1242/dev.202504 (PMC10906098; doi:10.1242/dev.202504)
Supplement: Supplementary information [file develop-151-202504-s1.pdf]

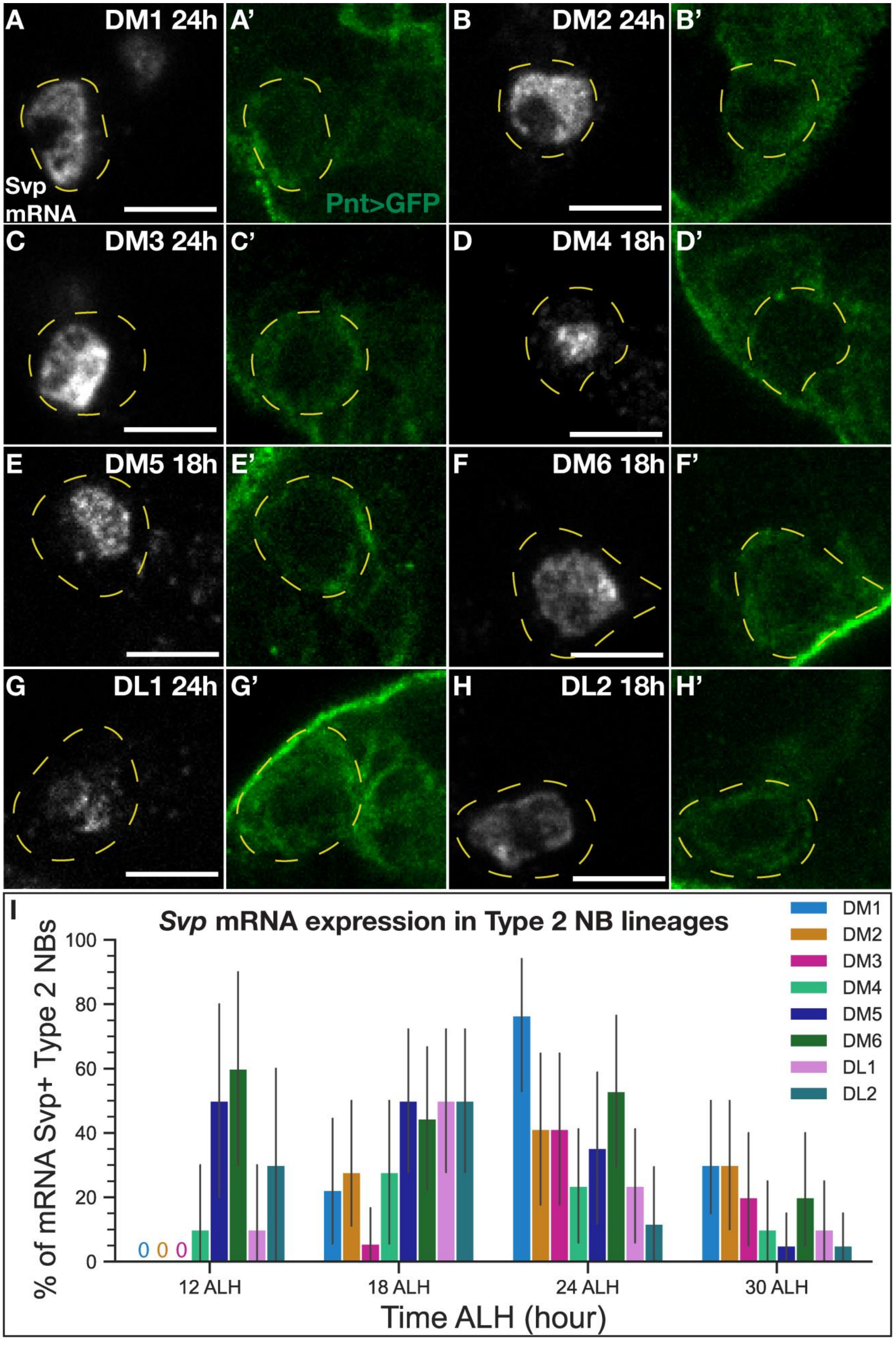

Development • Supplementary information

Fig. S1. Svp mRNA is expressed early in all larval T2NB lineages similar to protein expression.

(A-H) In all images, Svp mRNA is in white and T2NBs identified with Pnt-Gal4>GFP. Svp mRNA is detected at high levels in the T2NB nucleus and at very low levels in the cytoplasm, possibly due to our probe labeling a large intronic region of the svp RC isoform. Dashed yellow lines, T2NB. (A-C',G-G') Svp mRNA is expressed at 24h after larval hatching (ALH) in T2NB lineages DM1-3 and DL1. (D-H') Svp is expressed at 18h ALH in T2NB lineages DM4-6 and DL2. (I) Quantification of Svp mRNA expression in T2NBs across 12h-30h ALH shown as a bar plot with 95% confidence interval. For each lineage, 12 ALH, n = 10; 18h ALH, n = 18; 24h ALH, n = 17; 30h ALH, n = 20 lobes. Scale bars: 5 μm.

Development • Supplementary information

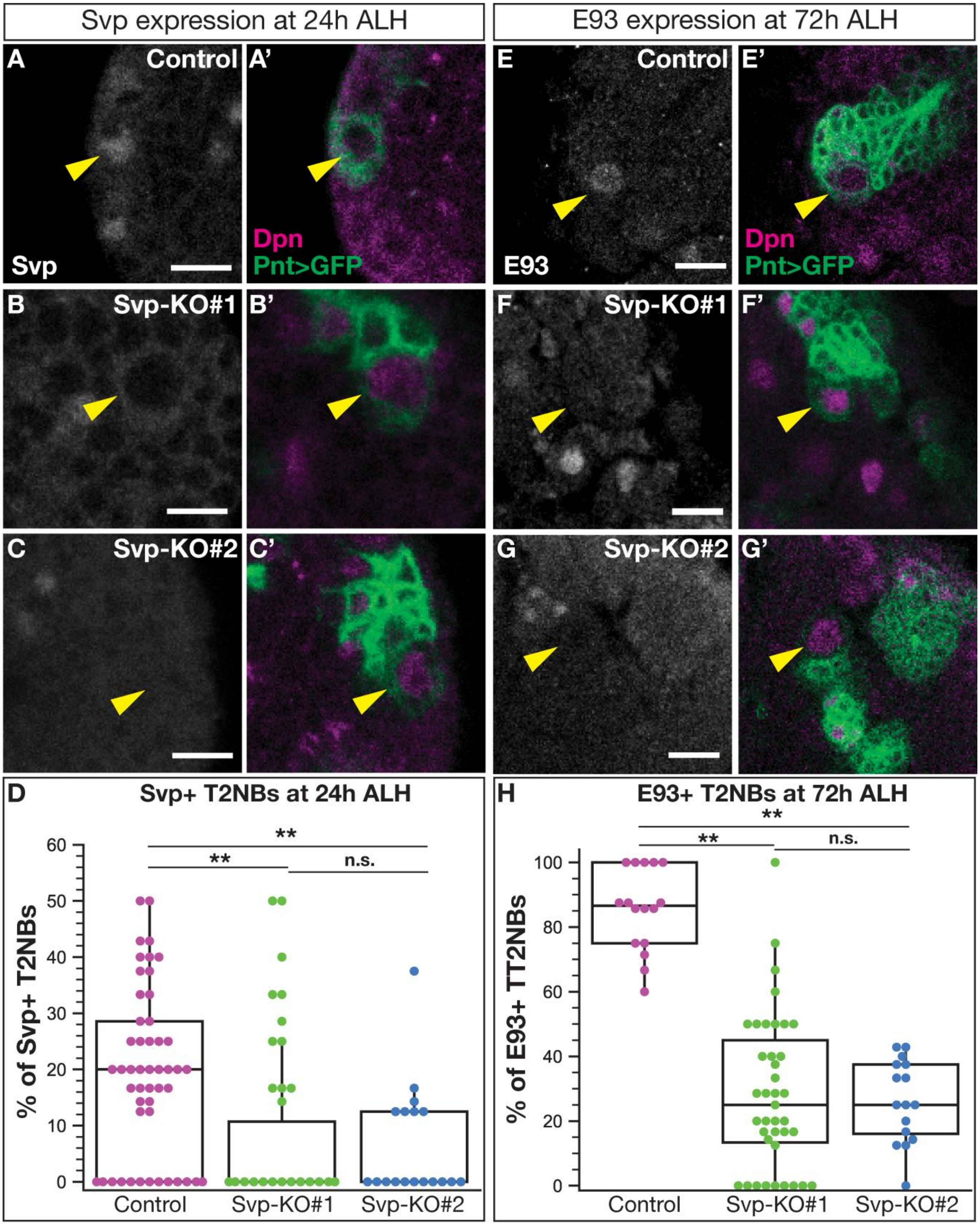

Development • Supplementary information

Fig. S2. Svp CRISPR/Cas9 knockout Svp in T2NBs and prevents temporal expression of the late factor E93.

(A-C') Svp-KO reduces occurrence of Svp in 24h after larval hatching (ALH) T2NBs. (D) Quantification of Svp expression in T2NBs at 24h ALH. Each dot represents one larval brain lobe with box and whisker plot showing distribution. Control = 49, Svp-KO#1 = 46, Svp-KO#2 = 18 lobes. *P*-values determined by One-way ANOVA, $P < 0.001$, with Tukey post-hoc test: Control versus Svp-KO#1 $P < 0.001$, Control versus Svp-KO#2 $P = 0.004$. (E-G') Svp-KO reduces occurrence of E93 expression in T2NBs at 72h ALH. (H) Quantification of E93 expression in T2NBs at 72h ALH. Each dot represents one larval brain lobe with box and whisker plot showing distribution. Control = 16, Svp-KO#1 = 39, Svp-KO#2 = 16. *P*-values determined by One-way ANOVA, $P < 0.001$, with Tukey post-hoc test: Control versus Svp-KO#1 $P < 0.001$, Control versus Svp-KO#2 $P < 0.001$. In all panels, transcription factor of interest is in white and T2NBs identified with Pnt-Gal4>GFP and Dpn. Scale bars: 5 μm.

Development • Supplementary information

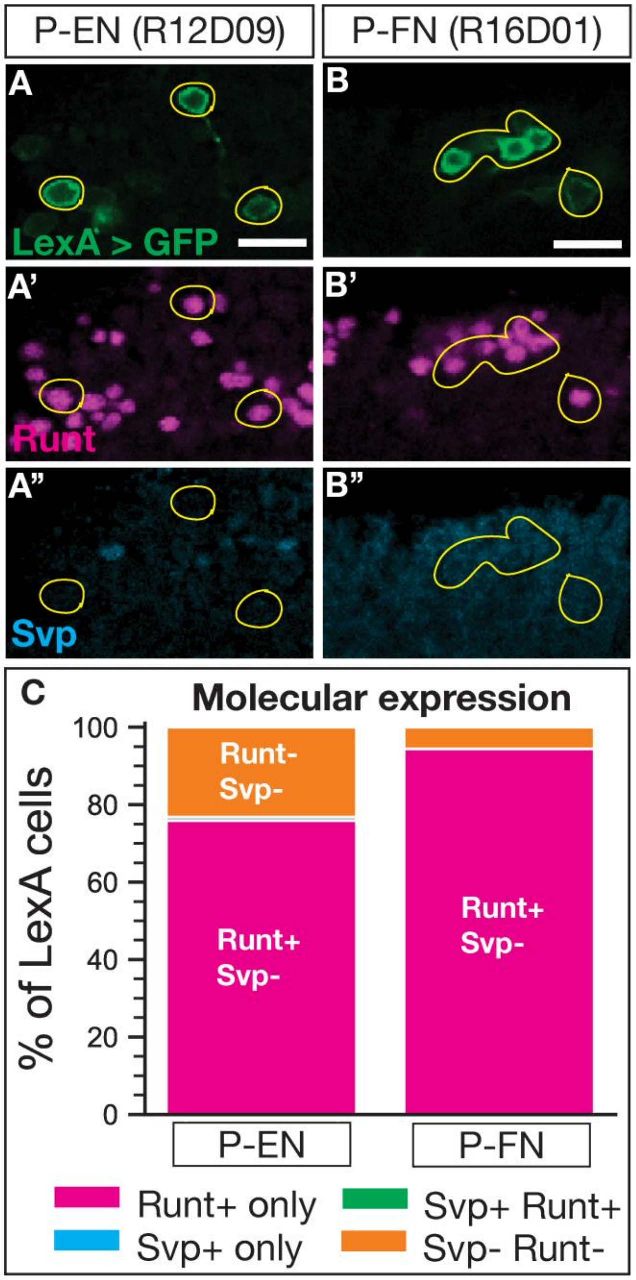

Fig. S3. Adult P-EN and P-FN neurons do not express Svp.

(A-B'') P-EN and P-FN neurons labeled by LexA driver lines express Runt but do not Svp. (C) Quantification of molecular expression in P-EN and P-FN neurons. P-EN and P-FN, n = 6 brains. In all panels, LexA+ neurons in green, Runt in magenta, and Svp in cyan. Yellow outline, neurons of interest. Scale bars: 10 μm.

Development • Supplementary information

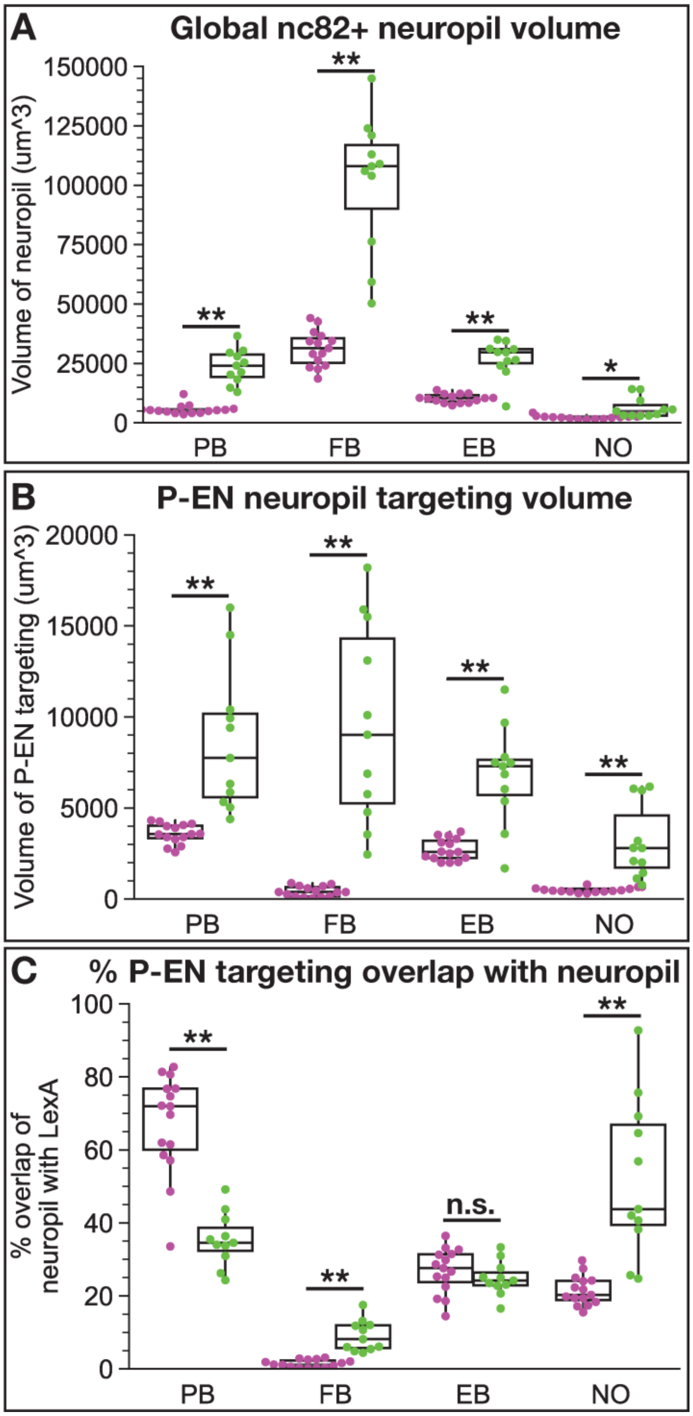

Fig. S4. Svp in T2NBs regulates adult CX neuropil development.

(A-C) Quantifications of neuropil volumes between Controls and Svp-KO#1. All plots show each dot representing one adult brain with box and whisker plot showing distribution. For all panels, Control, n = 15; Svp-KO#1, n = 11 brains, and *P*-values determined by independent t-tests. (A) Quantification of CX neuropil volume (μm^3) from

Development • Supplementary information

nc82 reconstruction. Control versus Svp-KO#1 for PB $P < 0.001$, for FB $P < 0.001$, for EB $P < 0.001$, for NO $P = 0.012$. (B) P-EN LexA neuron targeting volume (μm^3) to CX neuropils. Control versus Svp-KO#1 for PB $P = 0.002$, for FB $P < 0.001$, for EB $P < 0.001$, for NO $P < 0.001$. (C) Percent of CX neuropil volume (μm^3) targeted by P-EN LexA neurons. Control versus Svp-KO#1 for PB $P < 0.001$, for FB $P < 0.001$, for EB $P = 0.34$, for NO $P < 0.001$.

Table S1. Transgenes and *Drosophila melanogaster* stock lines used.

Genotype	Source and identifier	Additional information
R12D09-LexA	BDSC #54419	Expressed in P-EN neurons
R16D01-LexA	BDSC #52503	Expressed in P-FN neurons
13xLexAop-myr::GFP	BDSC #32210	Expresses membrane bound GFP under LexAop control
10XUAS-IVS-myr::GFP	BDSC #32198	Expresses membrane bound GFP under UAS control
Pointed-Gal4	*PMID:* 22143802 *14-94*	Expressed in Type 2 lineage starting in the neuroblast
10xUAS-IVS-myr::smGdP::HA, 13xLexAop2-IVS-myr::smGdP::V5	BDSC #64092	Expresses HA membrane tag under UAS control, V5 membrane tag under LexAop control
hsFLP; ;UAS-Cas9.P2	BDSC #58986	Expresses Cas9 under UAS control
hsFLP; UAS-sgRNA::svp ;	VDRC #341527	Expresses two short guide RNAs against Svp under UAS control; Svp-KO#1
hsFLP; UAS-sgRNA::svp ;	VDRC #341390	Expresses two short guide RNAs against Svp under UAS control; Svp-KO#2

Development • Supplementary information

Table S2. Genetic crosses for each experiment.

Figures	Summary	Genetic cross
Figure 1;	(i) Labels adult P-EN neurons<br>(ii) Labels adult P-FN neurons	Females containing *13xLexAop-myr::GFP* were crossed to males containing either (i) *R12D09-LexA* or (ii) *R16D01-LexA*
Figure 2;<br>Fig. S1	Labels larval Type 2 lineage	Self-cross of females and males containing *10XUAS-IVS-myr::GFP ; Pointed-Gal4*
Figure 3;<br>Fig. S3	(i) Labels adult P-EN neurons<br>(ii) Labels adult P-FN neurons	Self-cross of females and males containing (i) *10xUAS-IVS-myr::smGdP::HA, 13xLexAop2-IVS-myr::smGdP::V5; R16D01-LexA; Pointed-Gal4* or (ii) *10xUAS-IVS-myr::smGdP::HA, 13xLexAop2-IVS-myr::smGdP::V5 ; R12D09-LexA ; Pointed-Gal4*
Figure 4	(i) Control<br>(ii) Svp-KO#1<br>(iii) Svp-KO#2	Females containing *10xUAS-IVS-myr::smGdP::HA, 13xLexAop2-IVS-myr::smGdP::V5; R16D01-LexA; Pointed-Gal4* were crossed to males containing either (i) *hsFLP* or + ; *UAS-Cas9.P2*, (ii) *hsFLP* or + ; *UAS-sgRNA::svp* (VDRC #341527) ; *UAS-Cas9.P2* , or (iii) *hsFLP* or + ; *UAS-sgRNA::svp* (VDRC #341390) ; *UAS-Cas9.P2*
Figure 5;<br>Figure 6;<br>Supplement Figure 4	(i) Control<br>(ii) Svp-KO#1<br>(iii) Svp-KO#2	Females containing *10xUAS-IVS-myr::smGdP::HA, 13xLexAop2-IVS-myr::smGdP::V5 ; R12D09-LexA ; Pointed-Gal4* were crossed to males containing either (i) *hsFLP* or + ; + ; *UAS-Cas9.P2*, (ii) *hsFLP* or + ; *UAS-sgRNA::svp* (VDRC #341527) ; *UAS-Cas9.P2*, or (iii) *hsFLP* or + ; *UAS-sgRNA::svp* (VDRC #341390) ; *UAS-Cas9.P2*
Figure 7;<br>Fig. S2	(i) Control<br>(ii) Svp-KO#1<br>(iii) Svp-KO#2	Females containing *10XUAS-IVS-myr::GFP; Pointed-Gal4* were crossed to males containing either (i) *hsFLP ; ; UAS-Cas9.P2* , (ii) *hsFLP* or + ; *UAS-sgRNA::svp* (VDRC #341527) ; *UAS-Cas9.P2* , or (iii) *hsFLP* or + ; *UAS-sgRNA::svp* (VDRC #341390) ; *UAS-Cas9.P2*

Table S3. Antibodies used.

Antibody	Source and identifier	Additional information
Chicken anti-GFP	Aves: 1020	(1:1000)
Mouse anti-Seven-up 6F7	DSHB: Hiromi, Y. / Hondo, T. / Kanda, H.	(1:4)
Rat anti-Deadpan	Abcam: 11D1 BC7.1B	(1:20)
Guinea pig anti-Runt	Claude Desplan lab (NYU)	(1:1000)
Mouse -anti-Cut 2B10	DSHB: Rubin, G.M.	(1:10)
Mouse anti-V5 tag	ThermoFisher: R960-25 (previously Invitrogen 46-0705)	(1:1000)
Rabbit anti-V5 tag	Cell signaling: 13202S	(1:1000)
Rat anti-CadN DN-Ex #8	DSHB: Uemura, T	(1:50)
Mouse anti-nc82	DSHB: Buchner, E.	(1:100)
Rat anti-Imp	Claude Desplan lab (NYU)	(1:200)
Rabbit anti-Imp	Paul Macdonald	(1:500)
Rabbit anti-Syncrip	Claude Desplan lab (NYU)	(1:200)
Rabbit anti-pHH3	Millipore Sigma: MC463	(1:1000)
Mouse anti-pHH3	Abcam: 14955	(1:1000)
Guinea pig anti-Eip93	GenScript: 542604-33	(1:500)
Alexa Fluor® 488 AffiniPure Donkey Anti-Chicken IgY (IgG) (H+L)	Jackson ImmunoResearch, West Grove, PA: 703-545-155	(1:400)
Rhodamine Red™-X (RRX) AffiniPure Donkey Anti-Rat IgG (H+L)	Jackson ImmunoResearch, West Grove, PA: 712-295-153	(1:400)
Alexa Fluor® 647 AffiniPure Donkey Anti-Mouse IgG (H+L)	Jackson ImmunoResearch, West Grove, PA: 715-605-151	(1:400)
Rhodamine Red™-X (RRX) AffiniPure Donkey Anti-Guinea pig IgG (H+L)	Jackson ImmunoResearch, West Grove, PA: 706-295-148	(1:400)

Alexa Fluor® 488 AffiniPure Donkey Anti-Mouse IgG (H+L)	Jackson ImmunoResearch, West Grove, PA: 715-295-151	(1:400)
Alexa Fluor® 488 AffiniPure Donkey Anti-Rabbit IgG (H+L)	Jackson ImmunoResearch, West Grove, PA: 711-295-152	(1:400)
Rhodamine Red™-X (RRX) AffiniPure Donkey Anti-Mouse IgG (H+L)	Jackson ImmunoResearch, West Grove, PA: 715-295-151	(1:400)
Alexa Fluor® 405 AffiniPure Donkey Anti-Rabbit IgG (H+L)	Jackson ImmunoResearch, West Grove, PA: 711-475-152	(1:400)

Development • Supplementary information

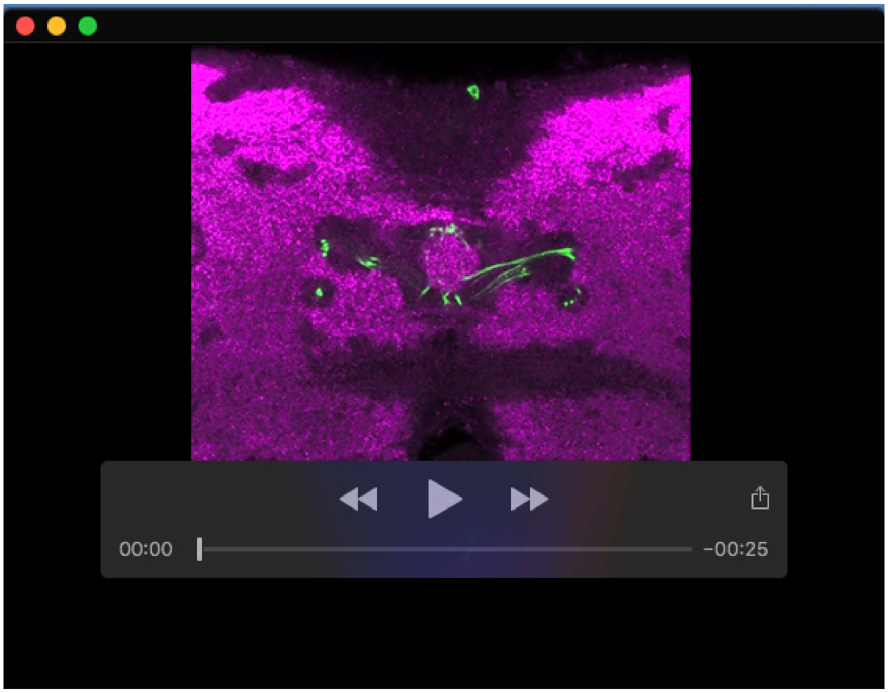

Movie 1. Control P-FN LexA neuron morphology in green and CX neuropils with NCad staining in magenta.

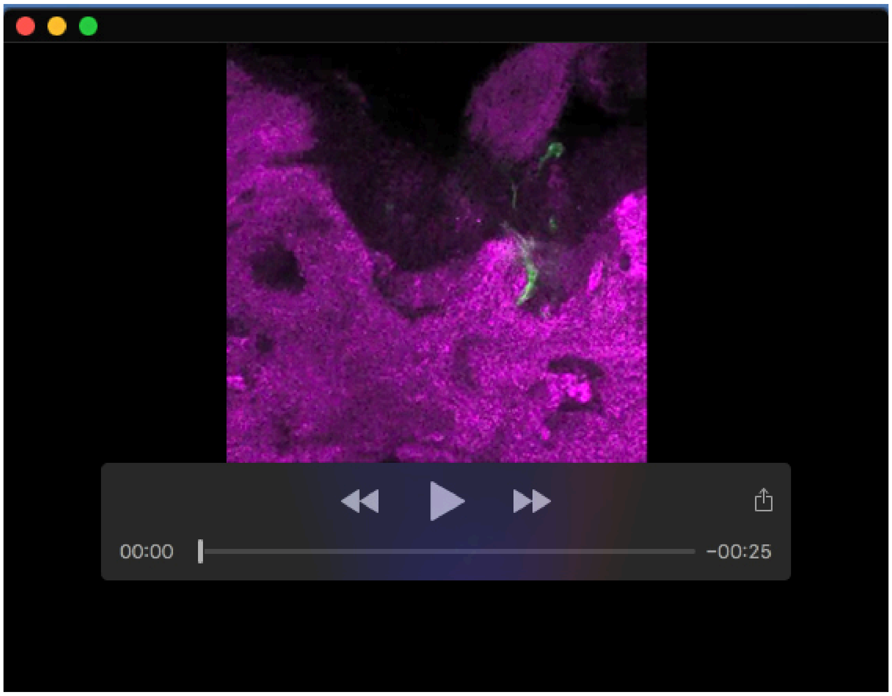

Movie 2. Svp-KO#1 P-FN LexA neuron morphology in green and CX neuropils with NCad staining in magenta.

Development • Supplementary information

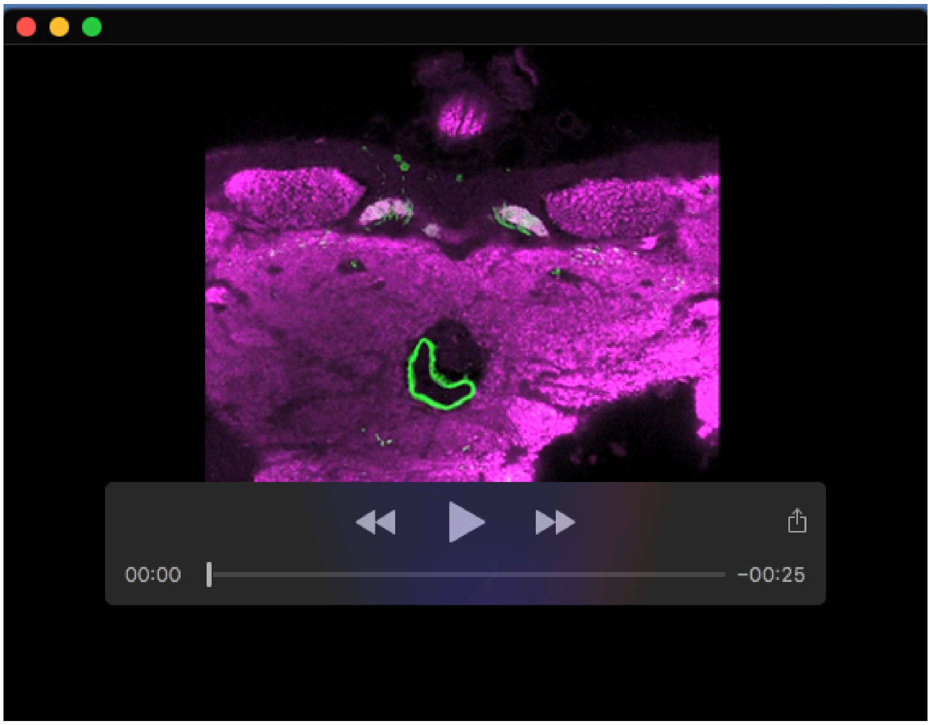

Movie 3. Control P-EN LexA neuron morphology in green and CX neuropils with nc82 staining in magenta.

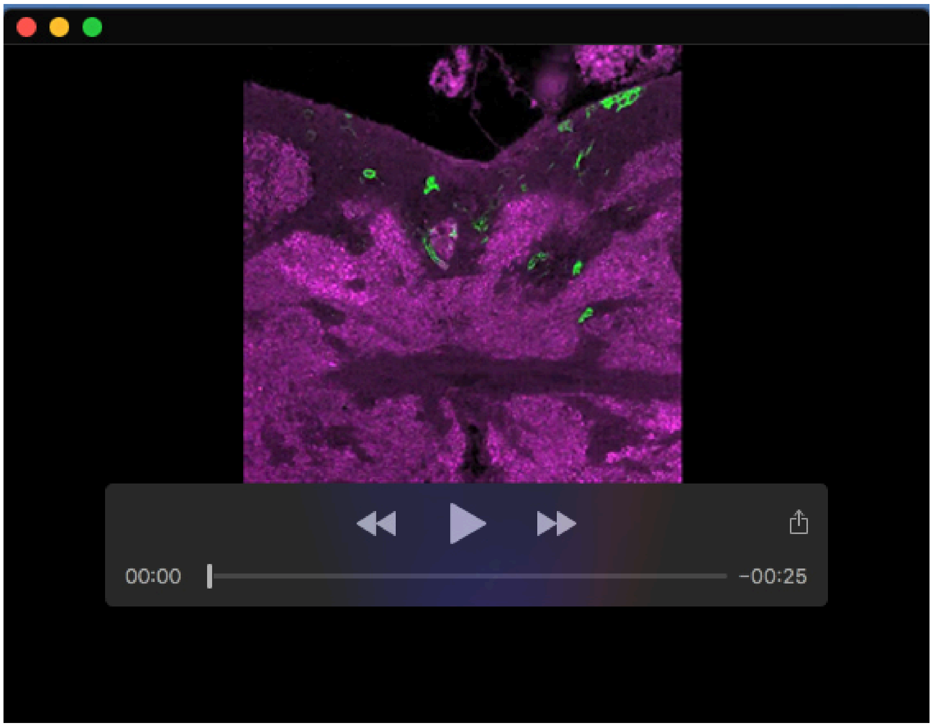

Movie 4. Svp-KO#1 P-EN LexA neuron morphology in green and CX neuropils with nc82 staining in magenta.
